# Low‐Dose Electron Total Scattering Analysis Resolves Non‐Crystalline Phase Separation in Polymer Semiconductors and Device Multilayers

**DOI:** 10.1002/smtd.70719

**Published:** 2026-05-17

**Authors:** Sang T. Pham, Adam F. Sapnik, Sean M. Collins

**Affiliations:** ^1^ Facility for Electron Microscopy School of Metallurgy and Materials University of Birmingham Birmingham UK; ^2^ Bragg Centre for Materials Research & School of Chemical and Process Engineering University of Leeds Leeds UK; ^3^ Department of Chemistry University of Copenhagen Copenhagen Denmark; ^4^ School of Chemistry University of Leeds Leeds UK; ^5^ Department of Materials Royal School of Mines Imperial College London London UK

**Keywords:** amorphous solid, analytical electron microscopy, crystallinity, crystallization, electron diffraction, focused ion beam, materials science, pair distribution function, polymer, semiconducting polymer

## Abstract

Nanoscale phase separation in polymer semiconductor blends significantly influences their mechanical, optical, and transport properties, and uncontrolled phase separation ultimately contributes to the long‐term degradation of devices. Recent advances in electron microscopy have enabled imaging and diffraction‐based analysis of polymer components, but these approaches are typically limited to blends with components exhibiting sharp differences in crystallinity or molecular structure. Here, we employ low‐dose scanning electron diffraction to characterize phase‐separated domains of components with nearly identical molecular structure, namely poly(9,9‐di‐*n*‐octylfluorenyl‐2,7‐diyl) (F8) and poly(9,9‐dioctylfluorene‐*alt*‐benzothiadiazole) (F8BT). For semicrystalline blends, we demonstrate phase identification and crystallographic texture analysis. In fully amorphous systems with partial phase separation, we highlight the limitations of electron pair distribution function (ePDF) analysis. Instead, we exploit differences in angle‐dependent scattering, coupled with calculated intramolecular scattering intensities, to reliably map distinct amorphous phases. Finally, we showcase this suite of techniques for characterizing a model device cross‐section, prepared by cryogenic focused ion beam milling. These workflows decouple phase separation and crystallization processes in F8:F8BT blends, provide corroborating insights into F8 crystalline and amorphous intermolecular π − π stacking, and support the direct visualization of non‐crystalline organic multilayer interfaces in cross‐section needed for failure analysis in organic optoelectronics.

## Graphical abstract

The performance of polymer blend semiconductors is governed by both the degree of phase separation and crystallization, but these two aspects have remained unresolved. We report methods for mapping amorphous‐amorphous as well as amorphous‐crystalline phase separation in polyfluorene‐based polymer blends using spatially resolved nanobeam electron total scattering analysis to examine phase distributions in both plan view and device cross‐section models.

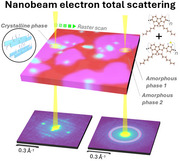

## 1 Introduction

Organic semiconductors (OSCs), and particularly systems incorporating polymer semiconductors, continue to break new ground across a range of applications harnessing new materials and photophysical properties for organic light emitting diodes (OLEDs) [1] for use in the commercial displays market as well as in organic photovoltaics now exceeding 20% efficiency [2], competitive alternatives to inorganic photodetectors [3], and transistors for flexible electronics [4]. A defining feature of polymer semiconductor devices is that they are multi‐component structures, both (i) in the separate organic device layers, i.e., the active absorber or emitter layers, electron transport layers, and hole transport layers, and (ii) within the layers, i.e., as polymer blends, bulk heterojunctions, or host–dopant emitter materials. Moreover, these materials exhibit a range of ordered and disordered states, forming semicrystalline and amorphous phases depending on the combination of materials and processing conditions applied. These features present a significant challenge for nanoscale characterization, the necessary first step in linking variations in molecular packing and crystallization, aggregation, or phase separation to performance across OSC devices.

Emission and absorption processes in OSCs are governed by excitonic states, that is, bound electron–hole pairs, but these events in the active absorber or emitter layers are also necessarily coordinated with charge transport from or extraction to the electrodes. Across a wide range of polymer blend and host–dopant emitter designs, a balance is needed between the long‐range, through‐device conductive pathways required for charge transport from the electrodes and the short distances required for charge transfer within the active layer involved in excitonic absorption or emission. This tension between charge transport and exciton formation is a central driver in the use of multiple components, aimed at decoupling these processes by enabling transport within a single‐phase network and charge transfer across a large interfacial area. However, this balance also establishes an optimal degree of phase separation: Excess aggregation or phase separation is deleterious to device performance across OSC device materials. Often, solution processing (spin coating and solvent choice) as well as heat treatments during film preparation or during ageing reduce the performance and lifespan of OLEDs.

Polyfluorenes present a prototypical example, with a significant body of research examining the causes and optimization of phase separation both laterally and vertically in blends of poly(9,9‐di‐*n*‐octylfluorenyl‐2,7‐diyl) (F8) and poly(9,9‐dioctylfluorene‐*alt*‐benzothiadiazole) (F8BT) [5, 6, 7, 8, 9, 10, 11, 12, 13, 14, 15, 16] as well as other F8BT‐based blends [17, 18, 19, 20, 21, 22]. Analogous aggregation effects are also a source of limited device lifespan in fullerene [23] and non‐fullerene [24] acceptor bulk heterojunctions for leading organic photovoltaics and photodetectors. F8:F8BT blends serve as an ideal model system for probing phase separation as they are designed to function as a green emitter through Förster resonance transfer from the blue‐emitting F8 to the green‐emitting F8BT, requiring an optimally homogeneous distribution for the required close contact. The optimal fraction of F8BT has been found to be 5% F8BT in F8 (19:1 F8:F8BT) for OLED device performance [9, 12, 25, 26, 27, 28], with crystallization of F8 occurring above 90°C [29]. Any deviation in composition or heat treatments have been shown to favor phase separation as reflected in increased blue luminescence [7, 13] and heat treatments introduce additional luminescence lifetime signatures of crystalline F8 [30]. As such, the occurrence of phase separation, both prior to and accompanying crystallization, and its effects on device performance are established. While these observations are pronounced when phase separation is advanced, the onset of such phase separation requires high resolution (nanoscale) detection.

Critically, crystallization can be distinguished from phase separation as phase separation can occur between two or more amorphous phases or may involve one or more crystalline phases. Nevertheless, the degree of ordering within or between phases often occurs concomitantly with aggregation and phase‐separation processes, particularly in thermally activated processes (heat treatments and ageing); that is, crystallization to a single‐component unit cell structure will often accompany and generally enhance phase separation. Consequently, while crystallinity in polymer OSCs generally tracks with improved charge transport [31], and high degrees of crystallization in poly(3‐hexylthiophene) (P3HT) have demonstrated drastic enhancements in characteristic length scales of energy transport [32], the control of crystallization (e.g., sequence of crystallization) rather than the overall degree of crystallization underpins performance gains [2]. Otherwise, crystallization occurring in tandem with phase separation and aggregation processes degrades device performance, as observed for rod‐ or fibril‐like crystallization accompanying phase separation in sub‐optimal F8:F8BT blends [8]. Taken together, a complete analysis requires nanoscale analytical probes capable of evaluating phase separation between multiple disordered and amorphous phases, as well as for monitoring crystallization processes in OSCs.

Phase separation processes in polymer‐based OSC materials have often been interrogated using scanning probe microscopy methods including atomic force microscopy (AFM) [7, 9, 10, 14], scanning near‐field optical microscopy (SNOM, with UV excitation transmission and fluorescence detection) [5, 8, 12, 33, 34], infrared spectromicroscopy (AFM‐IR) [35], and conductive probe measurements [16], though these methods are generally unable to examine changes across multiple layers in a device structure. Chemically sensitive imaging by energy‐filtered transmission electron microscopy (EFTEM) and scanning transmission electron microscopy‐based electron energy loss spectroscopy (STEM‐EELS) [36, 37, 38, 39, 40, 41, 42] has also been applied to visualize different electronic structure signatures arising from distinct phases in polymer blend OSC materials, offering depth‐resolved information in tomographic modalities. Depth profiling has also been reported using secondary ion mass spectrometry (SIMS) techniques [43, 44], neutron reflectometry [45], and ^3^He nuclear reaction analysis [10], and indirect information on phase separation can be extracted from time‐resolved spectroscopies [46, 47]. Resonant soft X‐ray scattering has also proven to be a powerful method for investigating phase separation in organic bulk heterojunction thin films [48, 49]. However, these approaches are unable to directly detect crystallization or examine changes across multiple layers in a device structure.

Crystallization has most often been examined using grazing‐incidence wide‐angle X‐ray scattering (GIWAXS), offering in‐plane and out‐of‐plane ordering analysis [50, 51, 52]. Low‐dose and cryogenic high‐resolution transmission electron microscopy (TEM) for lattice imaging and electron diffraction has also been applied to visualize the crystalline fractions and their spatial distributions in polymer OSCs and to infer key unit cell information or determine crystal structures [37, 53, 54, 55, 56, 57, 58]. Notably, for polymeric OSC materials, high‐energy electron or ion beams can irreversibly alter the structure through radiolytic beam damage, requiring careful control of electron fluence to minimize deviations from the native state of the material as a result of accumulated energy deposited in the sample (dose) [59]. These constraints on interactions with high‐energy beams have also presented challenges for device cross‐sectioning by focused ion beam (FIB) methods for analysis of vertical interfaces, ubiquitous in inorganic semiconductor device characterization but with limited exploration for OSCs to date [60, 61].

Emerging low‐dose four‐dimensional scanning TEM (4D‐STEM) techniques (also referred to as scanning electron diffraction, SED), where a two‐dimensional diffraction pattern is recorded at each probe position in a two‐dimensional raster across the sample at low electron fluence, further integrate nanoscale spatial resolution across micron‐scale fields of view while retaining rich crystallographic detail [62, 63]. These approaches have offered precise insights into the distribution of orientation in semicrystalline polymers [32, 64, 65, 66, 67] and how the crystalline distribution changes upon heating [68], yet these applications have primarily examined only the crystalline (diffracting) fraction. Recent advances in extending SED approaches for spatially resolved electron total scattering analyses, including electron pair distribution function (ePDF) analysis and imaging using angle‐resolved STEM [69, 70, 71, 72], now offer further prospects for probing phase separation in fully amorphous and mixed crystalline–amorphous OSC materials quantitatively.

**FIGURE 1:**
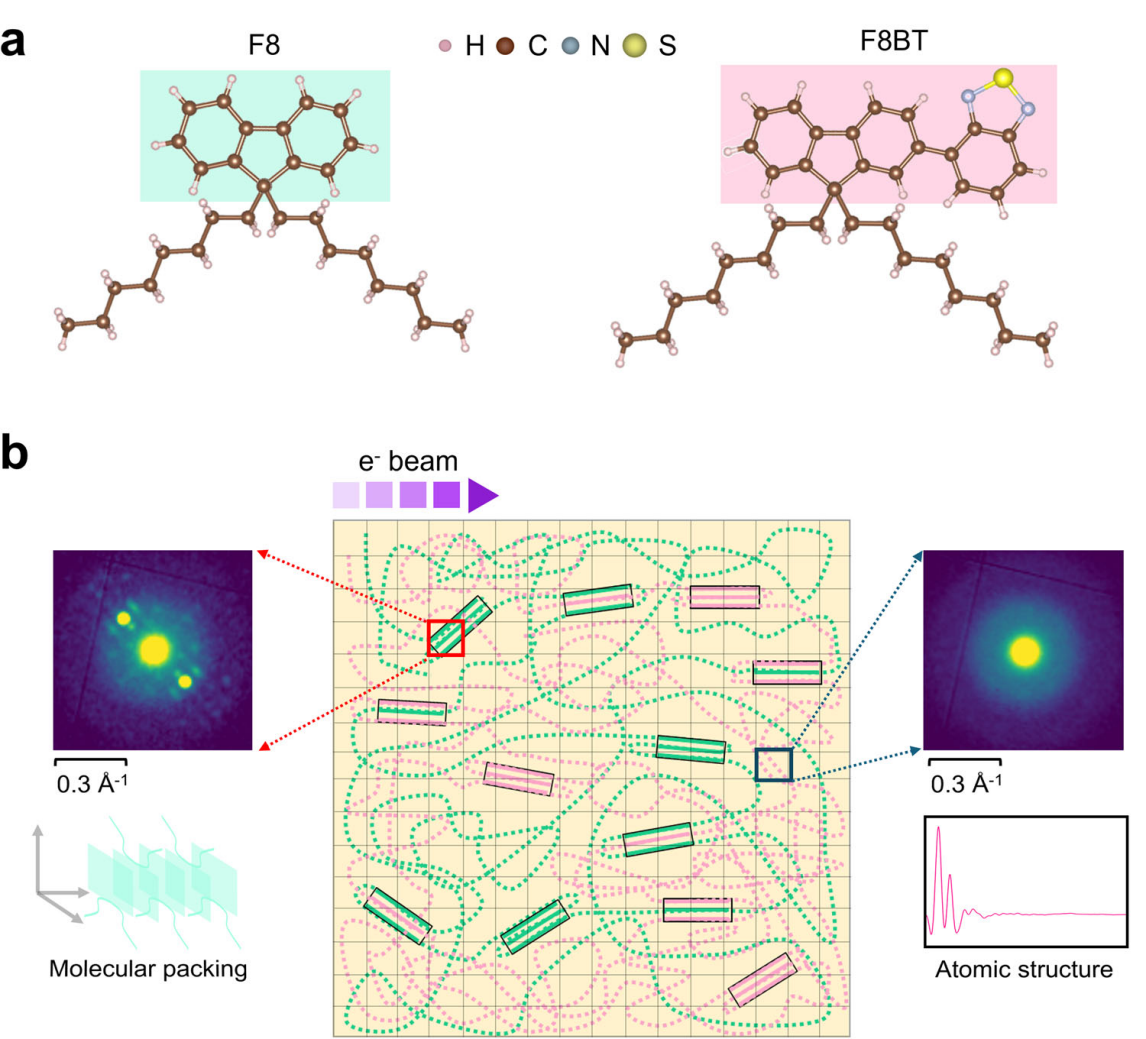
Chemical structure and packing in F8:F8BT blends. (a) Chemical structure of the monomers in F8 and F8BT polymers. (b) Schematic of SED imaging of an F8:F8BT semicrystalline blend. The black boxes highlight regions with ordered molecular packing. Bragg diffraction spots (left pattern) record the crystalline ordering, which is characteristically dominated by π–π stacking interactions within nano‐domains of F8 (green). Diffuse scattering (right pattern) encodes the disordered packing of F8BT (pink), which can be assessed using short‐range order analysis.

Here, we report an integrated methodology for examining amorphous–amorphous phase separation and crystallization in OSC plan‐view films and device cross‐sections, taking F8:F8BT blends as a highly suitable model system with sufficient prior study [5, 6, 7, 8, 9, 10, 11, 12, 13, 14, 15, 16] to serve as a benchmark (Figure 1). We select F8:F8BT as a particularly stringent model system because it is both a well‐studied benchmark blend in organic optoelectronics and a demanding test for diffraction‐based phase identification: the two polymers are chemically similar enough (Figure 1a) that distinguishing their amorphous phases is non‐trivial, while thermal treatment can also induce F8 crystallization. We demonstrate low‐dose SED total scattering analyses to image the onset of amorphous–amorphous and mixed crystalline and amorphous phase separation processes (Figure 1b). We record these processes as a function of blend composition and annealing temperature, with ePDF and angle‐resolved STEM observations explained by reference to Debye scattering equation calculations for the component monomers. In turn, we demonstrate a cryogenic thinning approach for FIB lift‐out to TEM, preserving crystalline polymers in device cross‐sections. SED total scattering analysis of cross‐sections likewise offers predictable contrast for distinguishing F8:F8BT and layers of the common hole transport material poly(3,4‐ethylenedioxythiophene) polystyrene sulfonate (PEDOT:PSS) in model multilayer structures. Our findings not only unveil the distinct phase separation and crystallization events in F8:F8BT, but also collectively, as methods, establish a robust framework for the characterization of phase separation and crystallization in isolated OSC layers through to OSC devices.

## 2 Experimental Section

### 2.1 Materials

Poly(9,9‐di‐*n*‐octylfluorenyl‐2,7‐diyl) (F8, powder, CAS: 19456‐48‐5), poly(9,9‐dioctylfluorene‐*alt*‐benzothiadiazole) (F8BT, fibers, CAS: 210347‐52‐7), and poly(3,4‐ethylenedioxythiophene) polystyrene sulfonate (PEDOT:PSS, 1:6 w/w, solution, CAS: 155090‐83‐8, 1.3 ‐ 1.7 wt.% solid content) were purchased from Ossila. Toluene (C_7_H_8_, CAS: 108‐88‐3, ≥ 99.8%) was purchased from VWR International. Acetone (C_3_H_6_O, CAS: 67‐64‐1, ≥ 99.5%) and 2‐propanol (C_3_H_8_O, CAS: 67‐63‐0, 70% in water) were obtained from Sigma‐Aldrich (Merck group). All chemicals were used as received. Deionized (DI) water (≥18.2 mΩ cm^−1^) was used for cleaning indium tin oxide (ITO) glass substrates and for floating the spin‐casted polymer films onto the TEM grids.

### 2.2 Sample Preparation for Freestanding Films

F8, F8BT, and their mixtures (19:1, 3:1, 1:1 wt./wt.) were prepared as thin films by spin‐coating. These blend ratios were selected to include the optimal ratio for green‐emitting OLEDs (5% F8BT, 19:1 F8:F8BT) [9, 12, 25, 26, 27, 73] as well as to map to specific benchmark reports on phase separation in 25% F8BT (3:1 F8:F8BT) blends [5, 7] and 50% F8BT (1:1 F8:F8BT) blends [8, 30]. Briefly, powders of F8, F8BT, and their mixtures were dissolved in toluene (at 40°C) to form solutions for spin‐coating. Prior to the coating of these emissive polymer layers, PEDOT:PSS (90 µL) was spin‐coated (3000 rpm, 60 s) on an ITO‐coated glass substrate (held at room temperature). Before spin‐coating, the ITO‐glass substrates were cleaned by sonicating in acetone, ethanol, 2‐propanol, and DI water sequentially for 15 min each. The substrates were dried at 80°C in an oven and then cleaned under Ar plasma for 10 min. Films of F8, F8BT, and their blends were spin‐cast onto substrates from a 7.5 mg mL^−1^ solution in toluene. The films were then spun at 3000 rpm for 45 s, followed by heat‐treatment at 80°C (5 min) to evaporate the solvent. Annealed films were further heated for 10 min at 150°C or 180°C, then cooled on a stone bench. These annealing times were selected to remain consistent with previous annealing studies in F8:F8BT based materials (e.g. 10 min annealing in devices [25]; longer annealing of 20 min at 150°C is known to induce substantial device performance losses [13]). Spin‐coating and annealing were performed under ambient conditions in air. Films were transferred onto lacey carbon TEM grids by dissolving the PEDOT:PSS layer in DI water, which resulted in the polymer semiconductor film floating onto the deionized water/air interface. The TEM grids were plasma‐cleaned for 10 s prior to the deposition of the polymer films. The residual water was allowed to evaporate at room temperature.

### 2.3 Sample Preparation for Cross‐Sectional Lamella

Site‐selective extraction of the model OLED structures was carried out using an FEI Helios G4 CX DualBeam focused ion beam (FIB) and scanning electron microscopy (SEM) system, equipped with platinum and iridium gas injection systems and a cryogenically cooled sample stage (Quorum). Prior to cross‐sectioning, a protective platinum‐carbon strip was deposited, with a total thickness of 1.2 µm; the first 200 nm was deposited using electron‐beam deposition, and the remaining thickness was deposited using ion‐beam deposition. Next, crude milling of the lamella was carried out. An approximately 3 µm cross‐section (thickness of the lamella, ultimately the specimen thickness in TEM/STEM) was then extracted and attached to a TEM half‐grid using a tungsten lift‐out needle. All of these initial steps were performed at ambient temperature. The TEM grid was then transferred to a cryo‐FIB shuttle. The shuttle was mounted on the cryo‐stage inside the FIB‐SEM chamber, which was gradually cooled to about −140°C by supplying liquid N_2_. The stage was then slightly tilted to align the TEM grid with the angle of incidence of the ion beam. A milling pattern was drawn around the region of interest, and the thinning was commenced using the parameters presented in Table 1. The thinning procedure was optimized from the paper by Gilchrist et al. [60], aimed at reducing the energy transfer perpendicular to the lamella. This reduction was accomplished by lowering the accelerating voltage, beam current, and angle which the beam makes with the lamella (denoted Φ in the table). After final thinning and cleaning, the cryo‐FIB shuttle was transferred into the sample transfer‐loading box using the transfer rod. The box was then vented and the shuttle (with the lid closed) was taken out, followed by natural heating to ambient temperature.

**TABLE 1:** Parameters used for thinning thick cross‐sections to electron‐transparent lamellas. The angle Φ represents the relative angle between the ion beam and the lamella. CCS refers to the cleaning cross‐section milling pattern (Thermo Fisher Scientific).

Lamella thickness (nm)	Accelerating voltage (kV)	Beam current (pA)	ɸ (°)	Milling pattern
∼1000 – 1500	30	790	±1.5	CCS
∼500 – 750	30	430	±1.5	CCS
∼250 – 350	30	250	±0.5	CCS
∼120 – 200	30	80	±0.5	CCS
∼100 – 150	30	40	±0.2	CCS
∼80 – 100	5	16	±0.2	CCS

### 2.4 Scanning Electron Diffraction (SED)

SED data were collected using a JEOL ARM300CF instrument (ePSIC, Diamond Light Source, UK) equipped with a high‐resolution pole piece, a cold field emission gun, aberration‐correctors in both the probe‐forming and image‐forming optics, and a 4‐chip MerlinEM (Medipix) pixelated electron‐counting STEM detector. The instrument was operated at 300 kV. For nanobeam diffraction, the aberration corrector lens elements in the probe‐forming optics were deactivated, and the condenser lens system was adjusted to achieve a convergence semi‐angle of 0.8 mrad using a 10 µm condenser aperture. This setup produces a 3 nm diffraction‐limited probe diameter *d_diff_* = 1.22λ/α at 300 kV for an electron de Broglie wavelength *λ* (1.97 pm at 300 keV electron energy) and convergence semi‐angle *α*. The probe current was set at ∼2 pA, calibrated using a Faraday cup with the beam passing through vacuum only, and the exposure time at each probe position was set to 1 ms. The estimated electron fluence for a single scan was approximately 17.6 e^–^ Å^−2^, assuming a disk‐like probe with a diameter equal to *d_diff_*. All SED measurements were conducted over a scan array of 256 × 256 probe positions. Calibration data for imaging and diffraction, including corrections for elliptical distortion in the diffraction plane, were acquired using a gold diffraction cross‐grating with a 500 nm period (Ted Pella). These cross‐gratings contain traces of Pd (AuPd), and calibration accuracy in Å^−1^/pixel calibrations for the camera lengths used was estimated to have an error margin of ∼1%, derived by comparison with a pure evaporated Au sample when calibrating with respect to the pure Au crystal structure (with no effect on elliptical distortion calibration). To minimize these effects on our analysis, complete pattern matching was applied for indexation (see Data Processing and Analysis). MoO_3_ crystals (Agar Scientific) were used to calibrate the relative rotation between the detector (diffraction pattern) and scan array axes, acquired under the same conditions as for the polymer samples. When accounting for this calibration, the diffraction patterns are rotated, resulting in a rotation of a ‘cross’ arising from a gap of 5 pixels in the readout electronics between the four quadrants of the MerlinEM (Medipix) detector.

### 2.5 Electron Energy Loss Spectroscopy (EELS)

STEM‐EELS and simultaneous high‐angle annular dark field (HAADF) STEM imaging data were obtained using a Thermo Fisher Scientific Titan Themis G2 microscope equipped with a continuously adjustable gun lens, multiple HAADF/ADF/BF STEM detectors, FEI Super‐X 4‐detector EDX system, a Gatan Quantum 965 ER spectrometer, and a Gatan OneView camera. The microscope was operated at 300 kV, with electron optics configured to achieve a convergence semi‐angle of ∼10 mrad, resulting in an estimated diffraction‐limited probe diameter of 0.273 nm. Core‐loss STEM‐EELS spectrum imaging (*K* and *L* edges) was performed using a spectrometer dispersion of 0.25 eV per channel. The spectrometer was operated in DualEELS mode to enable near‐simultaneous acquisition of the zero‐loss peak (ZLP) and plasmon energy losses, as well as the higher‐energy core ionization spectral window, facilitating accurate energy calibration of the core‐loss spectra. The collection semi‐angle was set to approximately 14.4 mrad for both low‐ and core‐loss measurements. To ensure efficient signal collection up to the oxygen *K* ionization edge (onset 530 eV), i.e., to >600 eV energy loss, the collection angle β should be such that β > α + 4θ_*c*_ or 14 mrad for a characteristic scattering angle θ_*c*_ taken as θ_*c*_ = *E_edge_*/(2*E*_0_) for incident energy *E_0_* [74]. This configuration provided coverage of a wide energy‐loss range, spanning 165–627 eV and effectively capturing the sulfur *L_23_* and carbon *K* edges. To minimize beam‐induced damage while balancing energy resolution and signal‐to‐noise ratio, the probe current was set to 45 pA, and a pixel acquisition time of 0.1 s was used. Spectrum imaging data were acquired with a pixel step size of 7.7 nm × 7.7 nm, yielding an acquisition fluence of approximately 4.7 × 10^3^ e^–^ Å^−2^ per pixel.

### 2.6 Data Processing and Analysis

SED data were analyzed, aligned, and calibrated using pyXem‐0.15.0 [75] in conjunction with supporting tools from the HyperSpy package (1.7.5) [76], following established methodologies described in prior studies [77]. The zero‐angle beam (direct beam) in each diffraction pattern was aligned by initially estimating the shift of the direct beam using a center of mass approach, followed by the alignment with the *align2D()* function in HyperSpy. The direct beam was then repositioned to the center of the pattern, and an affine transformation matrix was applied to correct the elliptical distortions and to eliminate the rotational offset between the SED raster pattern and the diffraction data. In some datasets, the orientation of the ‘cross’ pattern (caused by the gap in pixel readout between quadrants of the MerlinEM detector) differs across experiments due to variations in beam positioning across detector quadrants. ADF images were formed by integrating the diffraction pattern at each probe position (unless noted otherwise) using an inner radius of 0.12 Å^−1^ (2*θ* = 2.7 mrad) and an outer radius of 1 Å^−1^ (2*θ* = 22.4 mrad). These images are dominated by diffraction contrast because the angles span the Bragg angles at 300 keV beam energy. Average electron diffraction patterns for both entire and localized areas were obtained by taking the mean intensity from all diffraction patterns contributing to an image. Diffraction patterns are presented as the square root of the recorded intensity (applied in Fiji‐ImageJ software) for improved visualization of low‐ and high‐intensity features [78].

Crystallite orientation maps for the blends annealed at 150°C were generated using the approach of Chatterjee et al. [79]. Briefly, Bragg diffraction spots were detected by peak finding using a difference of Gaussians method. Filter settings were adjusted iteratively, with selected parameters verified by manual inspection to effectively capture disk‐like diffraction features in a randomized subset of diffraction patterns before applying the settings to the full four‐dimensional dataset. A map of the diffracting pixels (bright at the locations of crystalline domains) was created by plotting the number of found peaks at each probe position. An intensity threshold (a number of found peaks), selected after iteratively adjusting the threshold and checking the diffraction patterns recorded at locations above and below the threshold, was applied to label the pixels corresponding to diffracting components. For subsequent in‐plane orientation mapping of the diffracting domains, the Bragg pair associated with {600} planes in the reported F8 unit cell [80] was selected. The angular position of the Bragg pair was determined by radially integrating diffraction intensity over the range *k* = 0.20–0.30 Å^−1^, producing intensity as a function of azimuthal angle. The azimuthal angle spans the range − π to + π; we adopt a clockwise convention. Subsequently, the orientation corresponding to the highest intensity was extracted at each pixel to construct the orientation map. Prior to detecting local maxima, the azimuthal intensity profile for each diffracting pixel was smoothed using Gaussian filtering. The orientation values were folded back into the range 0 to π to account for the two‐fold equivalence arising from Friedel pair symmetry in diffraction, i.e., diffraction vectors **g**_hkl_ and − **g**_hkl_ (or $g_{\bar{h}\bar{k}\bar{l}}$) refer to an equivalent interplanar distance and planar stacking direction in the real space unit cell. We note that for multiple through‐thickness orientations (grains overlapping in projection), only the most intense orientation will be picked for such in‐plane orientation mapping. In cases where multiple through‐thickness domains are present, a series of virtual dark field (VDFs) images is better suited to display the data [32].

The orientation maps were further discretized into a set of angular bins of width *a* (*a* varies between 5–10° for the presented datasets), where *a* defines the maximum allowable orientation difference between neighboring pixels belonging to the same particle. For each orientation bin, contiguous groups of pixels were identified using an 8‐neighbour connected‐components analysis implemented in the open‐source Python package Scikit‐image 0.20.0. Regions smaller than three pixels were discarded to remove spurious detections. The resulting labelled regions correspond to individual crystallites, and the total number of labels was taken as the particle count within the analyzed field of view. For every particle, the pixel area, centroid position, and mean orientation were determined. The mean orientation for each region was calculated as a circular mean over all valid pixel orientations within the region, accounting for angular periodicity (0 equivalent to π). The distribution of particle orientations was visualized as a linear histogram of orientation frequency between 0 and π. Segmented maps displaying all identified particles were also generated to visually verify the accuracy of the segmentation.

Single‐crystal nano‐grains were assessed by manually inspecting diffraction patterns pixel‐by‐pixel in the four‐dimensional dataset. Sets of two or more spots in adjacent pixels at approximately the same orientation were used to identify a candidate single‐crystal domain. These were further validated by assessing the consistency in the number of spots, accounting for the possibility that certain reciprocal lattice points (corresponding to lattice planes) might tilt into or out of the Bragg condition, to verify that the overall spot pattern and orientation of the spot pattern continued across the candidate domain. The adjacent single‐crystal grain locations were then integrated to produce single‐crystal diffraction patterns for indexation, using the reported electron diffraction data for F8 and F8BT in the literature.

Particle size and morphology were analyzed using ParticleSpy [81]. Segmentation parameters were first examined and optimized using the *SegUI* interface to ensure consistent detection of crystalline grains across all datasets. A rolling ball background subtraction with a radius of 4–6 pixels was applied to enhance contrast between particles and the surrounding matrix. A Gaussian filter with a kernel size of 1 was used to smooth image noise prior to segmentation. Local Otsu thresholding was employed, using a local filter kernel size of 21 to define particle boundaries accurately. Watershed segmentation was activated to separate closely spaced grains, with the watershed seed separation parameter set between 2 and 3 pixels, and the seed erosion set to 1 pixel. A minimum particle size threshold of 4 pixels was applied to exclude noise and artefacts. The resulting segmented particles were quantified to obtain size distributions and morphological descriptors such as equivalent circular diameter and area for subsequent statistical analysis.

Experimental and calculated ePDFs were carried out following an approach as outlined previously [70, 72]. Briefly, azimuthal integration of area‐averaged experimental diffraction patterns was performed using pyFAI within pyXem. Before the integration, the direct beam disc was masked. Multiple and inelastic scattering contributions were treated by fitting the unstructured scattering profile at a high scattering angle, and an additional fourth‐order polynomial was included in the fitting procedure after fitting the atomic scattering profile. The scattering profile was then extrapolated to zero at the cut‐off imposed by the masking of the direct beam disc. A decaying exponential function, expressed as exp(−*bs* ^2^) where *s* denotes the scattering vector, and *b* is an adjustable parameter (here *b* = 0.25), was applied to adjust the scattering profile to zero at the maximum scattering angle before Fourier transform calculation. Simulation of ePDF for monomer units of F8 and F8BT was calculated using the Debye scattering equation, with electron scattering factors derived from the parameterization by Lobato et al. [82]. A *Q*_max_ value of 9 Å^−1^ was used in these simulations. The distributions of atomic distances for pairs of atoms (e.g., C‐C, C‐H, C‐N, C‐S, and N‐S) for the monomer units of F8 and F8BT were extracted using CrystalMaker software.

EELS spectrum images were processed using HyperSpy 1.7.5 [76]. First, the ZLP was aligned at 0 eV, and corrections were applied across both the low‐loss and core‐loss spectra. Background subtraction was performed by power‐law fitting between 120 and 137 eV. Following background removal, the spectra were cropped to the 120–270 eV range. This range captures the lowest energy used in the background fitting window before the sulfur *L_23_* edge, with a first onset expected at an energy loss of 165 eV. The sulfur map was then generated from the stripped *L_23_* edge by summing the signal over the energy window from 150 to 220 eV. Energy loss spectra in these areas were then extracted and normalized using the carbon *K* edge for comparative analysis. Specifically, the peaks at 285 eV (transitions to the π^*^ molecular orbital states) and 290 eV (transitions to σ^*^ orbital states) were used for normalization. Thickness maps were also generated using the log‐ratio (relative) method, which follows Poisson statistics. In this approach, the ratio of the intensity of zero‐loss electrons *I*_0_ to the total transmitted intensity *I*_t_ gives a relative measure of the specimen thickness *t* in units of the local inelastic mean free path λ (*t*/λ = *–ln*(*I*_0_/*I*_t_)) [83].

## 3 Results and Discussion

### 3.1 Morphology of F8:F8BT Blends

**FIGURE 2:**
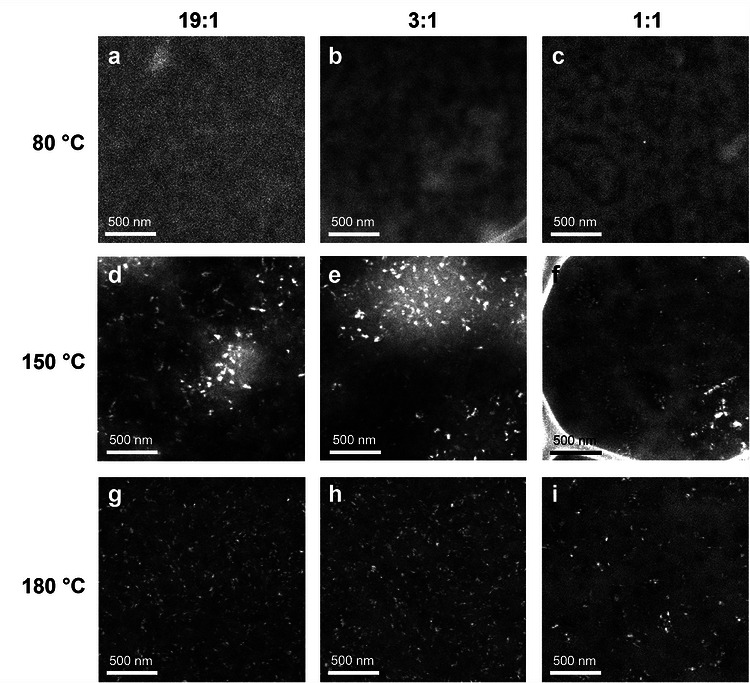
Morphology of F8:F8BT blends under the effect of blending ratio and temperature. (a–c) ADF images showing the morphology of 19:1, 3:1, 1:1 (wt./wt.) blends at 80°C, respectively. (d–f) Annular dark field images showing the morphology of 19:1, 3:1, 1:1 (wt./wt.) blends at 150°C, respectively. (g–i) Annular dark field images showing the morphology of 19:1, 3:1, 1:1 (wt./wt.) blends at 180°C, respectively.

Figure 2 shows the ADF images reconstructed from four‐dimensional SED datasets for F8:F8BT blends prepared with different blending ratios (19:1, 3:1, 1:1 wt./wt.) and annealing temperatures (80°C, 150°C, and 180°C). The annealing time was fixed at 10 min to mimic annealing reported in device construction [25] while also limiting thermal treatments to a single variable. Our objective here is to establish and validate a SED‐based methodology for total scattering to progress SED analysis beyond crystalline materials, also to multiple, adjacent non‐crystalline polymer semiconductor phases. For the blends annealed at 80°C, the 19:1 blend exhibits a uniform morphology with no distinct features (Figure 2a). However, structural domains begin to form as the F8BT content increases to 25 wt.% (3:1) and become more pronounced at 50 wt.% F8BT (1:1) (Figure 2b,c). These domains may be related to the changes in surface structure in the F8:F8BT blends (3:1 and 1:1 wt./wt.) observed in previous studies by AFM, which reported domains of 200–300 nm in diameter protruding from the surface [5]. Near‐field scanning optical microscopy (SNOM) has suggested that these raised phases are F8‐rich material [5].

In conventional STEM imaging, thickness variations can influence ADF imaging contrast and are especially expected to dominate contrast at higher scattering angles. However, in SED, the ADF images are more susceptible to diffraction contrast (with increased intensity from material diffracting to low scattering angles). In this case, we rule out significant diffraction by examining average diffraction patterns extracted from the bright and dark areas in the 3:1 and 1:1 blends (Figures S1 and S2). The patterns show only diffuse rings without sharp features, characteristic of an amorphous structure. This suggests that the contrast difference is not related to crystallinity. Nevertheless, contrast arising from differences in elastic scattering may not be due to simple thickness variation; thickness variation assumes consistent elastic scattering, an assumption which is not satisfied if significant changes in molecular structure or molecular packing are present. Regardless of the origin of the observed amorphous feature formation with increasing F8BT content, we note that (as in previous studies) any domain formation and associated phase separation comprises phases that are not fully separated, pure homopolymer domains, and instead are varying blends of F8 and F8BT [6]. We revisit the precise origin of the SED contrast after first considering crystallization phenomena arising from higher temperature treatments.

Upon annealing at 150°C and 180°C, distinct grain‐like features were observed within the films. These grains exhibit a high degree of crystallinity, as evidenced by the sharp diffraction rings of several orders observed in the average diffraction patterns (Figures S1–S3). The findings also demonstrate that the low‐dose conditions used for SED acquisition do not induce amorphization in the films that do not exhibit diffraction. At 150°C, the 19:1 and 3:1 blend films contain nanocrystalline grains exhibiting a rod‐like, elongated morphology (100–200 nm in length) intermixed with smaller, irregularly shaped grains, with generally smaller particles visible in the 1:1 blend (Figure 2d–f). These observations are borne out in particle size analysis, highlighting a decreasing grain size with increasing F8BT content (from 19:1 to 3:1 to 1:1) reflected by a decreasing equivalent circular diameter (40.1, 37.9, and 30.8 nm, respectively) for the associated crystalline domain areas (Figure S4). In the 19:1 and 3:1 blends, the grains are distributed evenly throughout brighter and dark regions of the film; in contrast, in the 1:1 blend, they are more localized and aggregated near or within low‐contrast regions (Figure 2f). At 180°C, the localization of crystalline domains persists in the 1:1 blend (Figure 2g–i), although differences in grain morphology and size distribution among the blends become negligible (Figure S5). These SED analyses reveal the microscopic crystallization behind the re‐appearance of blue emission (from F8) on thermal annealing in otherwise green‐emitting F8:F8BT materials [13].

The morphology changes observed on annealing are consistent with a temperature‐activated increase in molecular rearrangement that enables F8 crystallization within an already partially phase‐separated blend. At 80°C, the 3:1 and 1:1 films show amorphous domain formation without detectable Bragg diffraction, whereas at 150°C and 180°C distinct nanocrystalline grains emerge with sharp diffraction features. The glass transition of F8 is believed to be ca 50–80°C [11, 84, 85] whereas the glass transition in F8BT occurs above 100°C [11, 86], suggesting F8 is sufficiently mobile to re‐organise during annealing and undergo crystallization on cooling (F8 crystallisation at 90–100°C) [11, 85]. In our data, the stronger formation of the crystalline grains in the F8‐rich 19:1 and 3:1 blends, together with their indexation to the F8 unit cell, indicates that thermal treatment primarily promotes crystallization of the F8 component, as explained in earlier studies inferring crystallisation from spectroscopic signatures [30]. In the 1:1 blend, the crystallites are smaller and are localized predominantly near the interfaces between bright and dark domains rather than uniformly throughout the film, suggesting that the phase‐separated morphology constrains crystal growth and that F8‐rich regions adjacent to F8BT‐rich regions act as preferred sites for F8 nucleation and growth.

From ADF image analysis alone, it is not possible to unambiguously assign the nanocrystalline grains to either F8 or F8BT. However, the more extensive formation of nanograins in the 19:1 and 3:1 blends, i.e., those with F8 as the dominant component compared to the 1:1 blend annealed at 150°C, suggests that these grains are most likely F8 crystallites. Moreover, ADF image analysis of crystal distribution is limited by overlapping contrast contributions from thickness, non‐crystalline phase separation, and crystal orientation and size, prompting a closer inspection of the pixel‐by‐pixel diffraction data in the complete SED datasets summarized here.

### 3.2 Phase Identification and Orientation Analysis of Temperature‐Induced Crystallization

SED enables the investigation of a large field of view by diffraction using a nanoscale electron probe. For materials with large unit cells and finely spaced reciprocal lattice points (e.g., those with lattice parameters >20 Å as for F8 with *a* = 25.6 Å, *b* = 23.4 Å, and *c* = 33.2 Å) [80, 87], the flat Ewald sphere characteristic of high‐energy electrons will readily satisfy the Bragg condition for arbitrary crystal orientations within scattering angles corresponding to 1 Å^−1^ [72, 88]. Consequently, SED diffraction patterns for such materials typically display a number of diffraction spots within this scattering range. These spots, arranged in characteristic symmetrical patterns, reflect the crystal structure and enable phase identification by pattern matching and indexation to known unit cells. For F8:F8BT blends, SED enables the extraction and indexing of average diffraction patterns for individual grains within the field of view by examining the diffracting pixels located in or adjacent to the grain areas. Moreover, the location of crystalline and non‐crystalline regions within the field of view can be mapped (see Experimental Section). By combining real‐space information on crystallite morphology and the reciprocal space structural information, SED further offers an unambiguous determination of orientation relationships between diffracting grains and the crystallite growth direction [32, 65].

**FIGURE 3:**
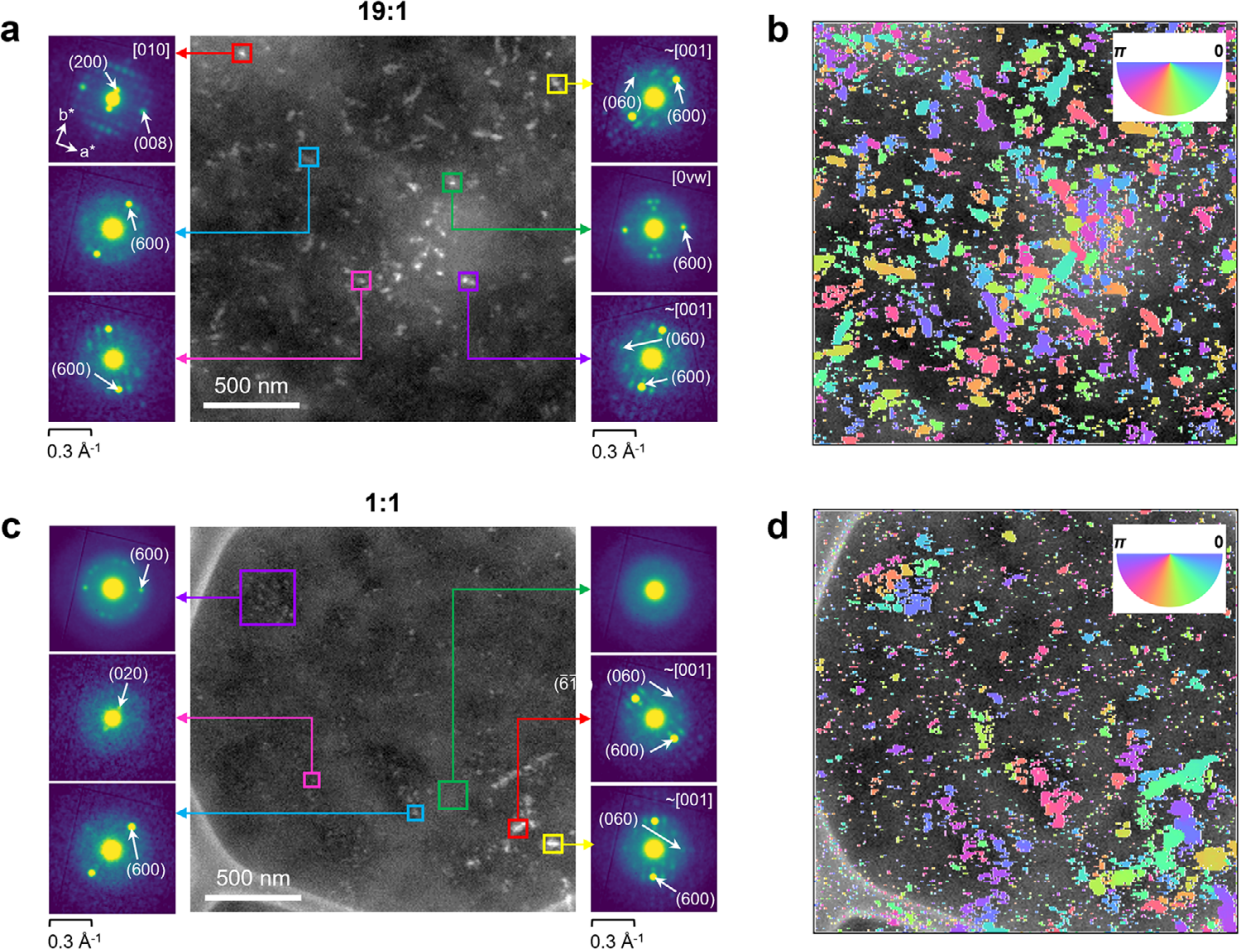
Single‐crystal diffraction pattern and crystallite orientation map of F8:F8BT blends annealed at 150°C. (a) ADF image and single‐crystal diffraction patterns of individual grains from a 19:1 blend film. (b) Overlay of the orientation crystallite map on the ADF image of the 19:1 blend film. (c) ADF image and single‐crystal diffraction patterns of individual grains from a 1:1 blend film. (d) Overlay of the orientation crystallite map on the ADF image of the1:1 blend film.

Figure 3a presents ADF images of 19:1 blend films annealed at 150°C, along with average diffraction patterns extracted for individual grains. Since this blend predominantly consists of the F8 phase (∼95 wt.%) and the diffracting grains are distributed randomly and evenly across the film, the grains are likely crystallites of F8. This claim is supported by indexing the single‐crystal diffraction pattern extracted from the grain marked in red (top left, Figure 3a). The pattern was indexed by measuring the *d*‐spacings of two prominent sets of diffraction spots and their relative angle. These measurements were then compared to previously reported diffraction patterns for F8 [80, 87]. The systematic rows of (*h*00), (0*k*0), and (00*l*) spots were extracted from the previous studies and overlaid on the zone‐axis diffraction pattern to verify the indexing. Following this procedure, the bright diffraction spots nearest to the direct beam were indexed to the (200) and ($\bar{2}$00) (d* = 0.085 Å^−1^, *d*‐spacing = 11.7 Å) reflections in the F8 unit cell, with a perpendicular set of bright spots indexed to the (008) and (00$\bar{8}$) reflections of the F8 unit cell (d* = 0.244 Å^−1^, *d*‐spacing = 4.09 Å). Consequently, the diffraction pattern of the grain in the red‐marked box corresponds to a view along the [010] zone axis, an orientation not previously reported experimentally but one that is self‐consistent with the previously observed orthogonal pair of [100] and [001] zones [80]. We note that the SED pattern agrees with the spot positions and the systematic absences expected for the (*h*00) and (00*l*) rows in the F8 unit cell [80].

Applying similar procedures, the brightest spots in diffraction patterns for other grains were indexed to the (600) and ($\bar{6}$00) interplanar spacings of the F8 unit cell. In some grains, marked in pink (Figure 3a, lower left), yellow (upper right), and violet (lower right), additional weakly diffracting spots could be indexed to the (610) and ($\bar{6}\bar{1}$0) or ($\bar{6}$10) and (6$\bar{1}$0) reflections of the F8 unit cell, indicating an orientation near the [001] zone axis. Patterns with fewer spots or discernible rows offered more limited indexation: For example, for the grain marked in green (Figure 3a, middle right), the brightest Friedel pair was readily indexed to the (600) and ($\bar{6}$00) spacings for the F8 unit cell (d* = 0.234 Å^−1^, *d*‐spacing = 4.27 Å), but the remaining pattern could not be fully indexed, leading to an assignment of the orientation as [0*vw*].

While the unit cell parameters of F8 have been reported [87], its crystal structure remains tentative [80]. Chen et al. [87] proposed an orthorhombic unit cell with the space group *P2_1_2_1_2_1_*, considering only packing between the conjugated backbones of F8 chains and the intrinsic helicity of this conjugated backbone. This model can differentiate between left‐ and right‐handed F8 chains in the unit cell. However, Brinkmann [80] later suggested that the crystallization of F8 is significantly controlled by the organization of *n*‐octyl side chains, which can enforce the planarization of the conjugated backbone. In other words, the possibility to observe either planar or helical conformations of the conjugated backbone depends on the structure of the alkyl side chains. Brinkmann proposed a tentative new space group of *Pnb*2_1_ for the F8 crystal, with improved modelling of π − π packing between conjugated backbones in a ‘dimer’ in the unit cell, i.e., with a* d*‐spacing of ∼4.4 Å and along [110] or [$\bar{1}$10] directions. This modelling is more reasonable than that proposed by Chen et al., showing the π − π packing along the [100] direction with a *d*‐spacing of ∼5.2 Å, a spacing that is likely too large to allow for significant π overlap between adjacent F8 chains. The space group *Pnb*2_1_ implies also one additional reflection rule, namely for the diffraction spots *h*0*l*: *h* + *l* = 2n, which appears to be obeyed in the [010] zone axis diffraction pattern (Figure 3a). Thus, our results appear to corroborate the Brinkman space group identification for the F8 crystals.

To further examine the crystallographic texture in the films, we used the commonly observed set of {600} diffraction spots from the diffraction patterns of individual grains to construct a map of the relative in‐plane orientations of the F8 crystallites. Figure 3b shows the resulting orientation map overlaid on the ADF image of the 19:1 blend. Analysis of the orientation distribution by counting the number of crystalline particles (Figure S6a) indicates an equal frequency across all in‐plane orientations, suggesting a random arrangement of nanocrystalline grains. The particle count exhibits possible peaks at orientations between 0–20°, 100–125°, and 150–175°, suggesting that these angular ranges may represent preferred stacking directions of F8 crystallites within the 19:1 blend, possibly arising from the elongated, anisotropic shapes of many grains. Visual inspection of the diffraction patterns and the major axes of several elongated particles confirmed that many such particles fall within these orientation ranges (Figure 3b). More detailed analysis of elongated domains shows that their long axes are oriented approximately 100–110° away from the plane normal of the (600) reflection—that is, across the direction from the direct beam to the (600) spot (Figure S7). In effect, the long sides of these grains appear to coincide with the {100} planes. When correlated with the indexation of the near–zone‐axis diffraction pattern (Figure S7, grain 4), this observation suggests that these particles may preferentially grow along the *b*‐axis (along [010] in the F8 unit cell).

The same orientation analysis was performed for the 1:1 blend, with average diffraction patterns extracted for individual grains (Figure 3c). These diffraction patterns predominantly exhibited indexable reflections corresponding to the (600) and ($\bar{6}$00) interplanar spacings. At the same time, several grains also showed additional reflections indexed to ($\bar{6}\bar{1}$0) (grains marked in red and yellow, Figure 3b, lower right) and (020) or (0$\bar{2}$0) (grain marked in pink, Figure 3b, middle left). Some of the diffraction patterns, particularly those extracted from the red‐ and yellow‐marked regions, could be indexed along the [001] zone axis. Figure 3d shows the corresponding crystallite orientation map constructed in the same manner as for the 19:1 blend and overlaid onto the ADF image. In contrast to the 19:1 blend, the diffracting grains appeared to be distributed much less uniformly across the field of view, instead exhibiting localization of crystalline grains only in select regions. These grains were predominantly located in the bright regions adjacent to the interfaces between bright and dark domains, corresponding to possible protruding and recessed areas, respectively, in the phase‐separated morphology. In comparison, few or no diffracting grains were observed within the dark regions or in the interior of the bright regions away from the interfaces (violet‐ and green‐marked boxes in Figure 3c).

Since the crystalline grains could be indexed to the F8 unit cell, this finding suggests that the bright regions likely represent F8‐rich domains (acting as a source of F8 for crystal nucleation and growth), consistent with previous reports [5]. These observations go further, indicating that the phase‐separated morphology promotes the preferential formation of F8 crystallites along the interfaces between F8‐rich and F8BT‐rich regions. The crystallites in the 1:1 blend exhibited more irregular shapes, were generally smaller in size (Figure S4c), and contained fewer elongated domains compared to those in the 19:1 blend. The orientation analysis indicates possible peaks in particle counts at 15–40° and 125–150°, a similar relative offset in histogram peaks of approximately 105–110° as for the clusters at 0–20° and 100–125° in the 19:1 blend. Notably, the in‐plane angles relative to the horizontal direction in the field of view are arbitrary, and so this similarity in the relative difference in preferred in‐plane orientations may suggest a common textural quality in the crystallization of the F8 crystallites during temperature‐activated phase separation.

### 3.3 Phase Mapping in All‐Amorphous Blends Using Diffuse Scattering

We now return to the question of STEM imaging contrast for amorphous phase separation observed in F8:F8BT blend films, the central target of this work, to demonstrate a method for non‐crystalline phase mapping. We use this aim to trace the initial phase separation that degrades device performance [7] and precedes temperature‐induced device performance losses [13]. The scattering data recorded in electron diffraction approximates total scattering (with reasonably complete orientational sampling) for non‐crystalline materials, making it well‐suited for ePDF analysis [69]. Electrons interact with matter much more strongly than X‐rays or neutrons, and the electron beam can be focused on a much smaller area, significantly reducing the required sample size. However, the instability of samples under the electron beam remains a key consideration [59]. Previous studies have demonstrated that SED acquisition supports spatially resolved ePDF analysis of MOFs and MOF‐glass composites under low‐dose electron exposure conditions [70, 72]. Here, we first explore the feasibility of the SED‐ePDF approach, focusing on the phase‐separated, 80°C‐treated 1:1 blend film (as in Figure 2c).

**FIGURE 4:**
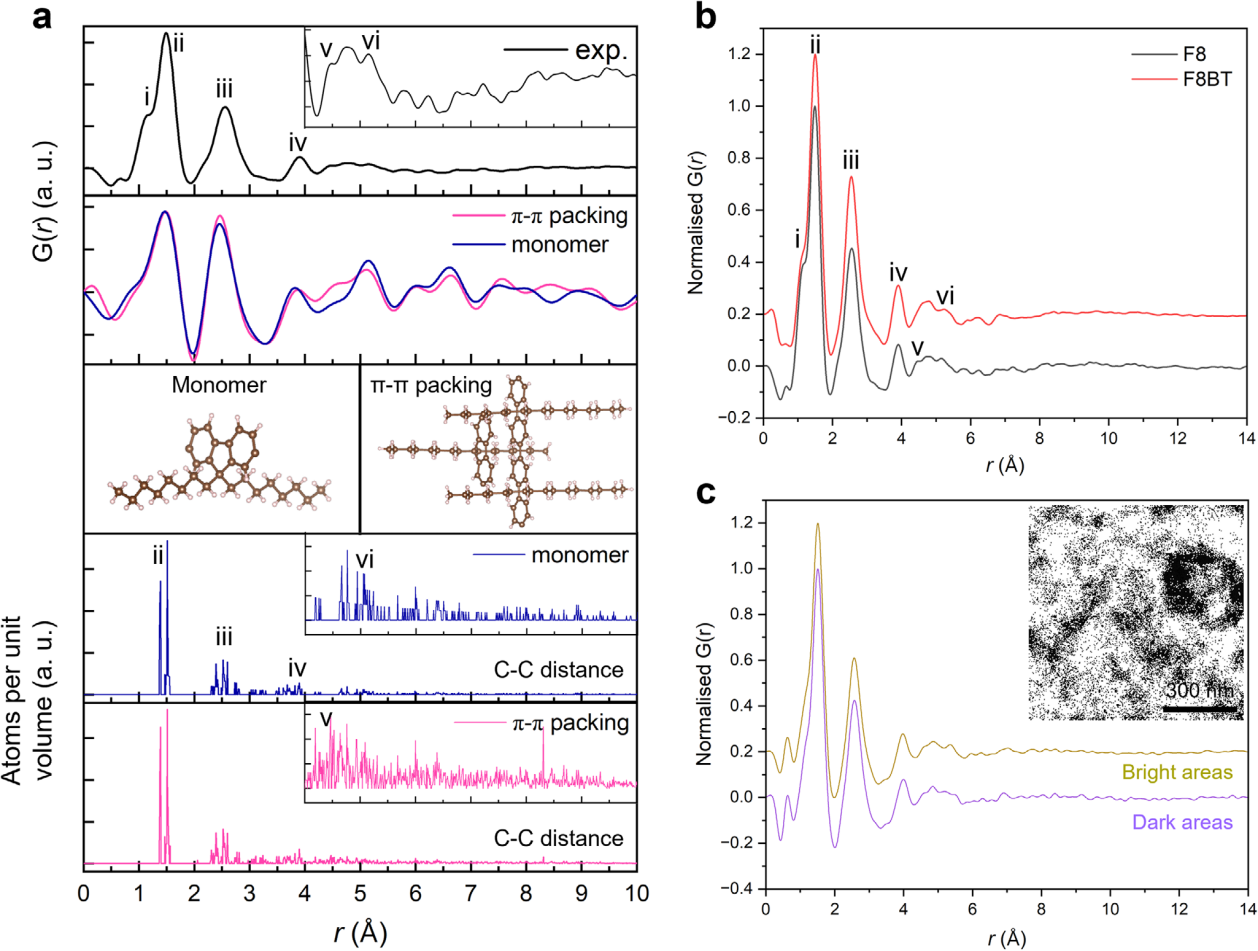
ePDF analysis of individual polymers and their 1:1 blend. (a) Experimental ePDF of an F8 polymer film (upper graph), alongside calculated ePDFs from the monomer (blue) and a molecular assembly including π − π stacking (pink, second upper graph). The molecular structures used for ePDF calculations, and the associated distributions of C‐C atomic distances extracted from the F8 monomer, are shown below. Insets show the magnified fine features from 4–10 Å. (b) Comparison between experimental ePDFs of F8 and F8BT polymers, data offset for clarity. (c) Comparison between ePDFs extracted from distinct amorphous fractions in the phase‐separated, 80°C 1:1 blend, data offset for clarity. These regions are denoted as bright and dark areas. The inset image shows a thresholded ADF image used to define these regions in the 1:1 blend film.

Before analyzing the blends, we applied ePDF analysis to films of each single‐component F8 and F8BT polymer under the same SED conditions. Figure 4a shows the ePDF, denoted *G*(*r*), obtained from an F8 polymer film. This film exhibits a predominantly amorphous structure (Figure S8a). To aid interpretation, we also calculated ePDFs for an isolated monomer unit and a two‐molecule assembly fragment incorporating π − π stacking. These calculations were carried out using the Debye scattering equation with Lobato electron scattering factors [82]. The two‐molecule assembly was built from a set of monomer units using the suggested π − π packing distance in the unit cell reported by Brinkmann [80]. An initial maximum scattering vector *Q*_max_ equivalent to that used in the measured ePDF (∼19 Å^−1^) was used. This maximum was then manually refined to an effective *Q*_max_ to match the peak broadening observed in the experimental ePDFs, which was found to be ∼9 Å^−1^. These calculated ePDFs represent a simple treatment of the short‐range order and may be limited due to unconsidered factors such as thermal motion effects, electron‐beam‐induced damage, or well‐known mismatch in ePDF peak intensities arising from multiple and inelastic scattering in experimental scattering data. The finite convergence angle of the electron nanobeam also reduces *Q*‐resolution, introducing a damping envelope at higher *r* in the final ePDF [70]. To minimize these effects, we rescaled the ePDF intensity to normalize the height of the first peak, preserving relative peak intensities and ensuring that peak positions remained robust against thickness variations. Although relative peak intensities were not directly comparable between the experimental and calculated ePDFs, the peak positions aligned closely, as shown in Figure 4a.

Crucially, the alignment of peak positions between experimental and calculated ePDFs for F8 (Figure 4a) confirms the retention of short‐range molecular structure during the electron exposure used for data acquisition. The calculated ePDFs further highlight stronger correlations for the π − π packing in the 1–6 Å range compared to the monomer unit (Figure 4a). Peaks at (i) 1.10 Å (C‐H intramolecular distances, Figure S9a), (ii) 1.48 Å, (iii) 2.54 Å, (iv) 3.90 Å, and (vi) 5.17 Å (C‐C intramolecular distances, Figure 4a) exhibit strong correspondence between the experimental ePDF and the ePDF calculated for the F8 monomer. However, the peak at (v) ∼4.41 Å likely arises from π − π packing, matching the corresponding π − π packing distance calculated for the two‐molecule fragment at 4.4 Å (Figure 4a; Figure S9b).

We also considered possible contributions from alkyl chain conformations, performing ePDF calculations for monomer units with varying upper‐limit rotations of alkyl chains (30°, 60°, 90°, and 120°). For each upper‐limit rotation, 1000 possible monomer structures were generated (Figure S10). The calculated ePDFs for the 60°, 90°, and 120° upper‐limit rotations show the emergence of the peak in the vicinity of, but not precisely aligned with, the observed peak (v) (Figure S10). Moreover, the peaks at (iv) and (vi) are not well‐aligned with the measured ePDF. We do note that the calculated ePDFs for 30° upper‐limit rotation offer improved alignment for all other peaks in the 1–6 Å range, albeit without a strong peak at the distance (v). Similarly, intramolecular rotation (0–180°) of the conjugated backbone was examined (Figure S11), as suggested by proposed helical backbone structures in F8 [89], but the calculated ePDFs exhibited significant mismatch with the experimental data. Consequently, these results reveal the presence of π − π packing within amorphous F8 phases in addition to that observed in the crystal structure.

The F8 ePDF profile enabled further comparisons with F8BT, shown in Figure 4b. The ePDF of F8BT, obtained from a diffuse ring‐dominated diffraction pattern characteristic of amorphous materials (Figure S8b), shows similar peak features at (i), (ii), (iii), (iv), and (vi). However, the peak at (v) is less pronounced in F8BT. Since F8BT contains a benzothiadiazole moiety, it is expected that the F8BT ePDF profile might show changes in peak positions, as suggested by the bond distribution distances of a F8BT monomer unit (Figure S12). Although the finite convergence angle of the electron nanobeam results in a damping envelope at high *r*, some atomic distances < 6 Å correlating to the C‐N, C‐S, H‐N, H‐S, N‐N, and N‐S pairs should still be well‐distinguished from C‐C and C‐H (Figure S12). However, as reproduced in our calculated ePDF for the F8BT monomer, the *G*(*r*) shows minimal differences in peak positions below 6 Å compared to F8 (Figure S13a), likely due to the low fraction of additional heteroatoms and the significant similarity of the majority of atoms in the monomer and their minimal differences in scattering factors. Calculations of the ePDFs for F8BT rotamers with varying backbone helicity (between the conjugated dioctylfluorene backbone and benzothiadiazole group [90]) also revealed no significant changes in peak positions below 6 Å (Figure S14). While high‐resolution, high signal‐to‐noise ePDF analysis may be able to distinguish pure, amorphous F8 and F8BT polymers, the distinguishing features are not sufficiently robust to identify partially separated phases in blends. Figure 4c shows the ePDFs extracted from the visually distinct bright and dark amorphous regions in the 1:1 blend. Peaks at positions below 6 Å are well reproduced and comparable to features observed in both F8 and F8BT.

One advantage of the SED technique is that the scattering datasets are recorded at individual probe positions during the STEM scan, enabling spatially resolved analysis. We performed a linear matrix decomposition using Principal Component Analysis (PCA) on the 3D data cube containing the ePDFs at each pixel in the scanned area of a 1:1 blend film (Figure S15a) to extract signals with high variance (see SI). Variance analysis indicated that two components offered a statistical representation of the data (Figure S15b). While the second principal component captured the typical form of an ePDF profile in its factorized *G*(*r*) component, other components show either non‐physical peaks or peaks unrelated to atom‐atom distances in their *G*(*r*) components; mapping the components offered no further insight (Figure S15c). Taken together, these findings indicate that this SED‐ePDF analysis is not sensitive to distinguish partially phase‐separated F8 and F8BT.

The ADF image (Figure 2c) nevertheless suggests phase separation is encoded in the SED data. These ADF images were created by integrating the signal within a specified annular range, from 0.12 Å^−1^ (2*θ* = 2.7 mrad) to 1 Å^−1^ (2*θ* = 22.4 mrad). This initial choice generates an ADF image exhibiting both thickness and structural information. Alternatively, the virtual ADF approach can be used to select narrow, successive angular ranges to produce angle‐resolved data, as suggested previously for mapping phase separation of P3HT and PCBM [69] and PS‐*b*‐PEO block copolymers [91]. However, in the P3HT/PCBM system, these features also offered distinguishable ePDFs [69], and phase mapping in the PS‐*b*‐PEO system relied on disentangling crystalline PEO signals [91]. Both systems rely on significant prior knowledge, a requirement that is not general and one that is not satisfied in the F8:F8BT blend system. Nevertheless, the approaches motivated the evaluation of angle‐resolved ADF imaging as an alternative approach to ePDF.

**FIGURE 5:**
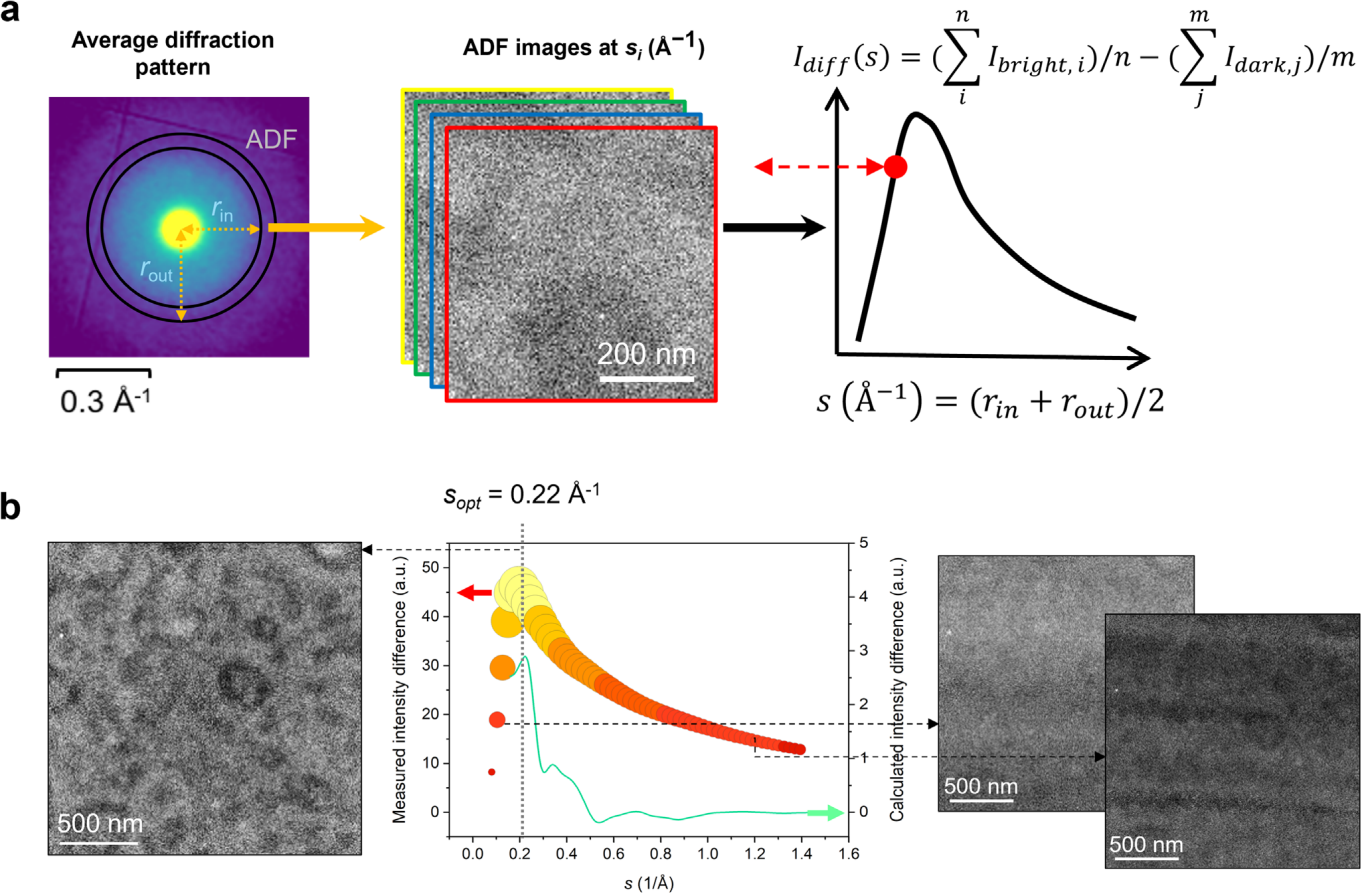
Contrast analysis of the ADF images formed at different annular ranges. (a) Schematic of the data analysis procedure showing how a series of ADF images was generated by systematically adjusting the annular range with the inner radius (*r*_in_) and outer radius (*r*_out_) of the virtual aperture. The intensity difference *I_diff_* between the bright and dark areas, as defined by the overall ADF image, is calculated for each ADF image within successive scattering‐angle ranges. *I*_bright, i_ is the intensity of pixel *i* in bright regions, *n* is the total number of pixels contributing to the bright regions, *I*_dark, j_ is the intensity of pixel *j* in dark regions, and *m* is the total number of pixels contributing to the dark regions. The measured intensity difference *I*_diff_ is plotted against the scattering distance *s*, defined as the average of (*r*_in_) and (*r*_out_). b) Measured (left, yellow‐red circles) and calculated (right, green line) *I*_diff_ profile for experimental dataset of the 80°C 1:1 blend. The left ADF image was extracted at the *s* showing the maximum in *I*_diff_ and unambiguously revealing the phase‐separated morphology. The right ADF images, extracted from the lower‐*s* and higher‐*s* regions, show reduced contrast.

To overcome the lack of prior knowledge for selecting a scattering angle for ADF imaging of phase separation in F8:F8BT, we develop two extensions: (1) We empirically retrieve a measure of contrast as a function of scattering angle, and (2) we also apply forward calculations of F8 and F8BT monomer structures to explain contrast variations. Figure 5a presents an overview of this workflow. Briefly, ADF images were generated by setting the inner radius (*r*_in_) and outer radius (*r*_out_) of the virtual aperture. To produce a sufficient signal‐to‐noise ratio in the ADF image, the radius integrated between *r*_in_ and *r*_out_ was set to 4 pixels. A series of ADF images were then automatically generated from successive scattering ranges defined by *r*_in_ and *r*_out_. The ADF image produced from a wide annular range from 0.12 Å^−1^ (2*θ* = 2.7 mrad) to 1 Å^−1^ (2*θ* = 22.4 mrad) was used as a map to identify bright and dark regions, and the intensity difference *I*_diff_ between the segmented bright and dark regions for each angle‐resolved ADF image was calculated as shown in Figure 5a. The angle‐resolved *I*_diff_ was then plotted against the representative scattering distance *s* (Å^−1^), defined as (*r*_in_, _i_ + *r*_out, i_)/2.

Figure 5b presents the *I*_diff_ plot as a function of *s* (Å^−1^) for the 80°C 1:1 F8:F8BT blend film, showing a maximum at *s* = 0.22 Å^−1^. The ADF image corresponding to the scattering range with the maximum in *I*_diff_ is shown on the left‐hand side of the plot, illustrating a clear observation of domain areas related to bright and dark regions in the phase‐separated morphology. ADF images corresponding to the lower *I*_diff_ at both lower and higher *s* (Å^−1^) show limited, if any, contrast for the phase‐separated domain structure. The narrow angular range giving a maximum in *I*_diff_ suggests that the contrast is not a simple mass‐thickness contrast, otherwise expected across all scattering angles, but is instead due to differences in elastic scattering from molecular structure or packing. Considering the coherent scattering intensities in reciprocal space to come from either intramolecular or intermolecular interferences, the reduced total scattering structure can be expressed as: *F*(*Q*) = *F_intra_* (*Q*) + *F_inter_* (*Q*). In amorphous materials, where the diffuse scattering dominates, *F*(*Q*) can be approximated to *F_intra_* (*Q*) [92]. In other words, the scattering intensity of molecular amorphous materials can be represented by the scattering signals from individual molecules, offering a route to the second extension for a forward calculation of diffuse scattering from F8 and F8BT monomers.

In (1:1) blend, F8 and F8BT coexist with approximately the same content, and so we hypothesized that the *I*_diff_ in real space could be determined by the interference of the scattering between individual F8 and F8BT molecules. To test our hypothesis, we performed Debye scattering calculations for individual monomer units of F8 and F8BT (Figure S16a). The difference between the scattering intensity of F8 and F8BT (Figure S16b) captures the scattering interferences between the monomers. As the convergence semi‐angle in SED is not zero, the scattered data in reciprocal space is properly convolved with the probe function, resulting in a reduction in *s* resolution (and *Q* resolution) [70]. Accordingly, we convolved the scattering intensity difference with a top‐hat function with a width equal to the size of the diffraction disc diameter (Figure S16c). The SED conditions used give a diffraction disc diameter of 2α/λ = 0.08 Å^−1^ (for λ at 300 kV and α = 0.8 mrad). Figure 5b (green plot) presents this experimentally‐matched *I*_diff_. Indeed, these calculations predict a maximum in *I*_diff_ at *s* = 0.224 Å^−1^, matching the experimental results in Figure 5b.

Correlating the experimental and calculated results in Figure 5b enables the identification of bright areas as F8 or F8‐rich phases, in agreement with previous studies [5] and our analysis of the growth of F8 nano‐crystallites at the edges of brighter regions (Figure 3). These data analysis procedures represent a significant step toward integrating readily calculated angle‐dependent scattering from structural fragments with empirical optimization of contrast. By using elastic scattering at low angles, our approach offers significant advantages over signals with weaker cross‐sections, such as elemental mapping by X‐ray energy‐dispersive spectroscopy or electron energy loss spectroscopy, which are particularly prone to complex contrast contributions from thickness and low signal variations in partially separated phases. We illustrate these challenges for sulfur mapping by EELS in Figures S17 and S18.

### 3.4 Cross‐Section Analysis of a Multi‐Layered, Model OLED Device With SED

The success of unravelling phase separation in freestanding F8:F8BT blend films prompted us to apply these approaches also to cross‐sectional analysis of a model device. We fabricated a simple model OLED multi‐layer stack comprising Glass/ITO/PEDOT:PSS/F8:F8BT (1:1), without the deposition of the top electrode. The hole transport PEDOT:PSS and emissive F8:F8BT layers were deposited on the ITO glass substrate via spin coating, according to a conventional device fabrication procedure for green‐emitting OLEDs. Cross‐sections of the multilayer stack were then prepared as a FIB lamella. A platinum capping layer was deposited on top of the multilayer stack to minimize the penetration of carbon and platinum nano‐particulates from the protective layers into the F8:F8BT emissive layer during FIB milling.

Final FIB thinning steps readily cause damage to polymer materials, and the damage mechanisms can be due to heat, implantation, and radiation damage [93] that can irreversibly alter the chemical structure and molecular packing. To minimize FIB damage, the final thinning steps were conducted under cryogenic conditions [94], and the voltage, beam current, and the angle the ion beams make with the sample were also optimized based on the reported FIB procedure for fullerene organic semiconductors [60]. Here, we deliberately selected the emissive layer as the 1:1 F8:F8BT blend, as this blend composition shows the most pronounced phase separation. We also intentionally annealed the device at 150°C to trigger the formation of the F8 nano‐crystallites. The choices served our purposes: (i) to verify if there is vertical phase separation in the emissive layer apart from the lateral phase separation observed in the freestanding film analysis; and (ii) to have well‐crystallized particles within the emissive layer to evaluate the damage caused by our FIB protocol.

**FIGURE 6:**
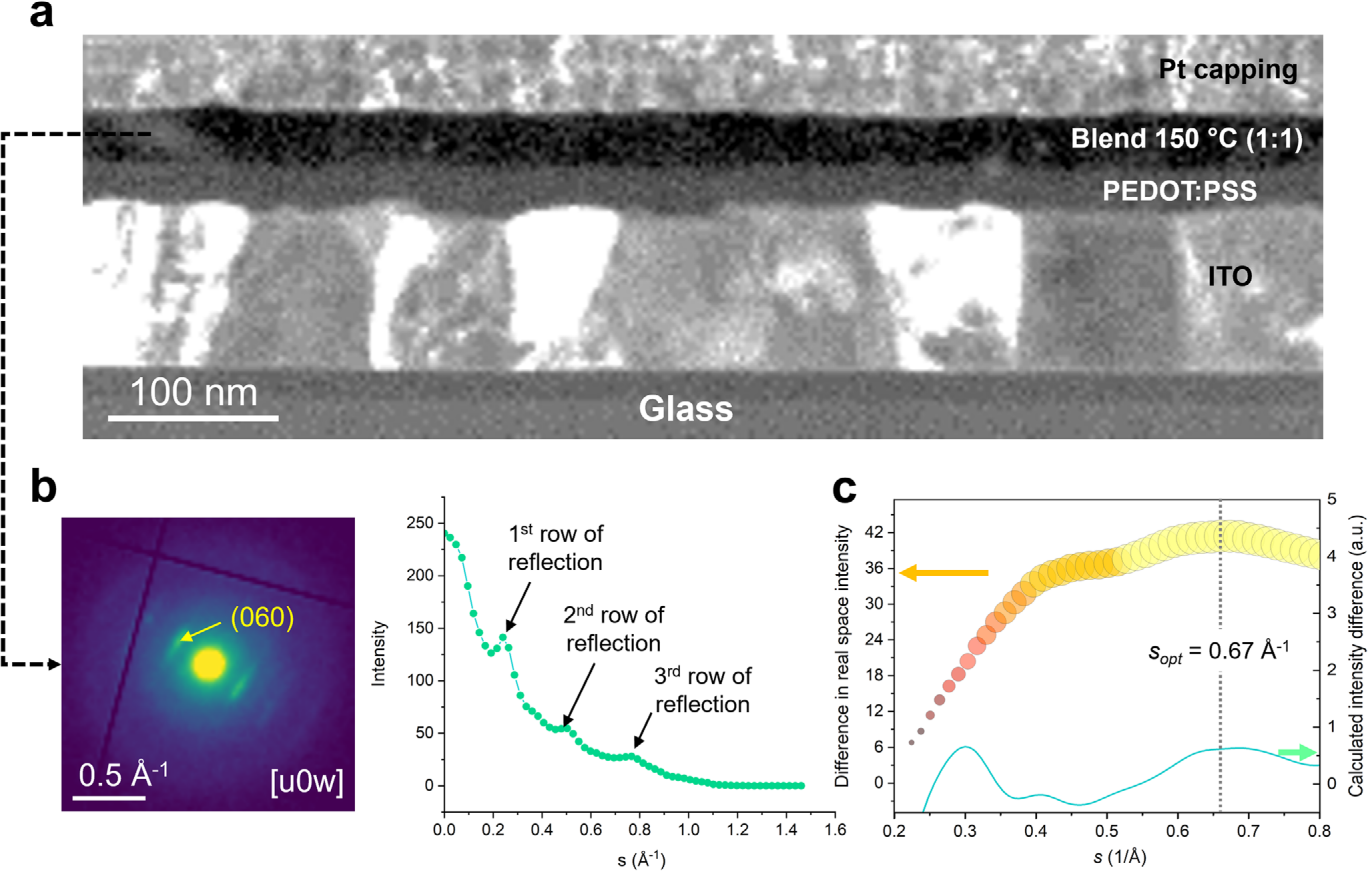
Demonstration of SED analysis on OLED model device cross‐section. (a) STEM ADF image of the OLED model device cross‐section. The brightness and contrast were adjusted to visualize two polymer layers (F8:F8BT (1:1) and PEDOT:PSS (1:6)) sandwiched between a Pt capping and an ITO‐glass substrate. Small and large domains can be observed within the F8:F8BT layers, and they could be F8 nanocrystals or Pt nanoparticles redeposited from the Pt capping during FIB milling. (b) Average electron diffraction pattern extracted from the large domain in the F8:F8BT layer shows the Bragg reflections of the finely spaced spots, typical of a material with a large unit cell (e.g., F8). The scattering intensity profile was plotted from the direction of the beam to high scattering distance *s*, showing the detectable second and third row of reflections for this particle. (c) Measured (left, yellow circles) and calculated (right, green line) *I*_diff_ profile via *s* for amorphous fraction of the cross‐section that contains the areas of both F8:F8BT and PEDOT:PSS blends. The ADF image in (a), corresponding to *s* at the *I*_diff_ maxima, unambiguously shows the significant difference in contrast between the two layers.

Figure 6a illustrates the ADF‐STEM image of the cross‐section prepared by the optimized cryo‐FIB technique. Two key observations stand out for the polymer layers: (i) Small and large particles are visible within the emissive layer, and (ii) a clear difference in contrast between the F8:F8BT layer and the PEDOT:PSS layer can be discerned. Figure 6b highlights the preservation of crystallinity after cryo‐FIB milling, showing a local average diffraction pattern extracted by SED with Bragg diffraction up to third‐order reflections in systematic rows. We indexed the intensity maxima for the first order reflection row as (060) based on the measured d* = 0.26 Å^−1^ (*d*‐spacing = 3.84 Å). Apart from this large domain, the remaining amorphous part of F8:F8BT layer exhibits uniform contrast, suggesting no vertical phase separation. The observation of Bragg reflections suggests that our cryo‐FIB technique successfully preserved the crystalline structure of F8 nanocrystals. Further evaluation of FIB damage in amorphous regions was performed using ePDF analysis (see Figure S19). The results confirm that there is no distinctively observable damage caused by either FIB milling or SED scanning on the internal molecular structure in either F8:F8BT or PEDOT:PSS blends.

Contrast between PEDOT:PSS and F8:F8BT layers does not come from diffraction contrast as they both exhibit amorphous structure (Figure S20a,b). As seen in F8 and F8BT, ePDFs did not show distinct differences at *r* lower than 4 Å, understandably as both polymers give rise to similar C‐C atomic distances (Figure S20c). We do not seek to analyze the ePDFs at *r* > 6 Å as this region appears to be dominated by typical ringing features arising from the limited signal‐to‐noise ratio (constrained also by the limited field of view for signal averaging) of the F8:F8BT and PEDOT:PSS layers in the cross‐section. To explain the contrast difference between the F8:F8BT and PEDOT:PSS layers, we now evaluate our angle‐resolved approach (Figure 5) for a second, distinct system. Figure 6c presents the angle‐dependent *I*_diff_ for the two layers. A broad peak with a maximum was identified unambiguously at *s* = 0.67 Å^−1^. For the calculation of scattering intensity of the blends (Figure S21), the angle‐dependent scattering intensity of individual components was first calculated, followed by linear combination according to the component ratio, i.e.*, I*_F8:F8BT_ = *I*_F8_+ *I*_F8BT_ and I_PEDOT:PSS_ = *I*_PEDOT_ + 6 × *I*_PSS_. After appropriately convolving with the convergence semi‐angle top‐hat function, the contrast function shows a maximum at *s* = 0.67 Å^−1^ (Figure 6c), aligning again with measured results. There is an additional local maximum at *s* = 0.29 Å^−1^, which we believe deviates from experimental data at low‐*s* due to strong multiple and inelastic scattering contributions at low angles (explaining a tendency toward zero contrast at low *s*) in the thicker lamella relative to the freestanding films. In any case, the scattering vector producing maximum contrast reproducibly offers a high degree of correspondence between experiment and fragment‐based calculation across both the plan view and cross‐sectional cases considered here, underlining the generality of the approach to angle‐resolved imaging of distinct amorphous polymer phases.

While we have considered a single set of film thicknesses here in plan view and in cross‐section, we note that the methodology established outlines several new avenues for investigation in F8:F8BT blends and also in other polymer blend films. For example, varying film thickness to probe thickness‐dependent effects on composition and thermally induced phase separation and crystallization processes follows logically from noted thermal property changes with variation in film thickness [85]. However, we note several constraints, requirements, and possible solutions for such studies in F8:F8BT or in other polymer blends, which may guide such future investigations. First, SED is fundamentally a projection‐based method, i.e. scattering signals for amorphous and crystalline (diffracting) phases are integrated from the entire volume along the beam trajectory. This creates at least two challenges, namely (i) multiple and inelastic scattering corruption of ePDF [70] and angle‐resolved scattering contrast and (ii) overlap of multiple, differently oriented crystals. Further work is required to establish whether recently proposed multiple scattering corrections [95] and energy‐filtering (to remove inelastic scattering) are viable in low‐dose SED configurations required to extend the presented methods to thicker plan‐view films. Additional steps to apply tilt‐series tomographic methods may then facilitate resolving features within film layers, albeit at a higher cumulative dose.

Alternatively, the cross‐sectioning methodology introduced here may support the evaluation of thicker films. Notably, the amorphous–amorphous phase mapping may be limited in quantification of amorphous structure by remaining ion‐beam surface damage to such lamellae. Evaluation of crystallinity, especially at low levels of crystallization, will also require the milling of many lamellae to ensure sufficient sampling through otherwise arbitrary slices from laterally extended film. The milling conditions reported here followed significant work to manage charging of the glass as well as evaluating room temperature and cryo‐milling conditions. Increased automation of such processing may offer routes to the necessary sampling for reliable evaluation of thicker films or for targeted, site‐specific lift‐out selected by correlation with other imaging modalities. These directions offer many opportunities to apply the methods presented here.

## 4 Conclusion

In this work, we report a workflow for the integrated analysis of amorphous‐amorphous phase separation and crystallization in semiconducting polymer films and cross‐sectional device stacks. Using F8 and F8BT as a model system, we showed that low‐dose SED can provide a comprehensive characterization of phase‐separated domains using both Bragg scattering and total scattering analysis of diffuse scattering data. In blends exhibiting semicrystalline structure, the indexing of Bragg diffraction from individual crystalline grains enables the phase identification and textural analysis of such nanocrystals. For solely amorphous blends where no Bragg scattering was observed, we note limitations for ePDF approaches: while ePDF confirms the retention of monomer structures in plan‐view F8 and F8BT films, the similarity of intramolecular and short‐range interatomic distances in only partially phase‐separated films limits real‐space distribution analysis. Instead, we show how angle‐resolved elastic scattering can be used to map phase separation, using Debye scattering fragment calculations to assign phases to the experimentally observed angle‐dependent scattering contrast. Our findings present a significant step toward understanding and predicting the diffuse scattering mechanisms of molecular materials and how these mechanisms can be harnessed to enable amorphous phase mapping, critical for unravelling nanoscale phase evolution processes and dynamics in devices across annealing during manufacturing through to operating conditions.

Finally, we demonstrated the capabilities of our developed technique and data analysis approach for cross‐sectional analysis of OLED model device stacks prepared by cryogenic FIB milling. We demonstrate the minimization of FIB damage on the active polymer semiconductors by establishing the preservation of crystalline Bragg scattering and then assessing the preservation of molecular (chemical) structure through ePDF analysis. Virtual ADF image analysis also enabled the interpretation of the contrast difference between two polymer semiconductor blends (F8:F8BT and PEDOT:PSS). These methods grant access not only to floated plan‐view films but also introduce a characterization capability approaching inorganic semiconductor device inspection of the crucial buried interfaces, now for organic electronics. Combined amorphous and semicrystalline phase analysis across these interfaces will offer needed insights into degradation and failure mechanisms in organic optoelectronic devices and, ultimately, will enable site‐specific interventions in fabrication and processing to improve their efficiency and operational durability.

## Conflicts of Interest

There are no conflicts of interest to declare.

## Supporting information




**Supporting File**: smtd70719‐sup‐0001‐SuppMat.pdf.

## Data Availability

The data that support the findings of this study (scanning electron diffraction and electron energy loss spectroscopy datasets) are available at Zenodo at https://doi.org/10.5281/zenodo.19882182.
