# Supplementary material for: Low‐Dose Electron Total Scattering Analysis Resolves Non‐Crystalline Phase Separation in Polymer Semiconductors and Device Multilayers
Source: Small Methods. 2026 May 17;10(11):e70719. doi: 10.1002/smtd.70719 (PMC13244434; doi:10.1002/smtd.70719)
Supplement: Supplementary file 1 — Supporting File: smtd70719‐sup‐0001‐SuppMat.pdf. [file SMTD-10-e70719-s001.pdf]

Low-dose electron total scattering analysis resolves non-crystalline phase separation in polymer semiconductors and device multilayers

Sang T. Pham[a,b], Adam F. Sapnik[c], Sean M. Collins[b, d, e*]

[a]Facility for Electron Microscopy, School of Metallurgy and Materials, University of Birmingham, Elms Rd, Birmingham B15 2SE, UK

[b]Bragg Centre for Materials Research & School of Chemical and Process Engineering, University of Leeds, Woodhouse Lane, Leeds LS2 9JT, UK

[c]Department of Chemistry, University of Copenhagen, Universitetsparken 5, 2100 Copenhagen Ø, Denmark

[d]School of Chemistry, University of Leeds, Woodhouse Lane, Leeds LS2 9JT, UK

[e]Department of Materials, Royal School of Mines, Imperial College London, Exhibition Road, London SW7 2AZ, UK

*Email: s.m.collins@imperial.ac.uk

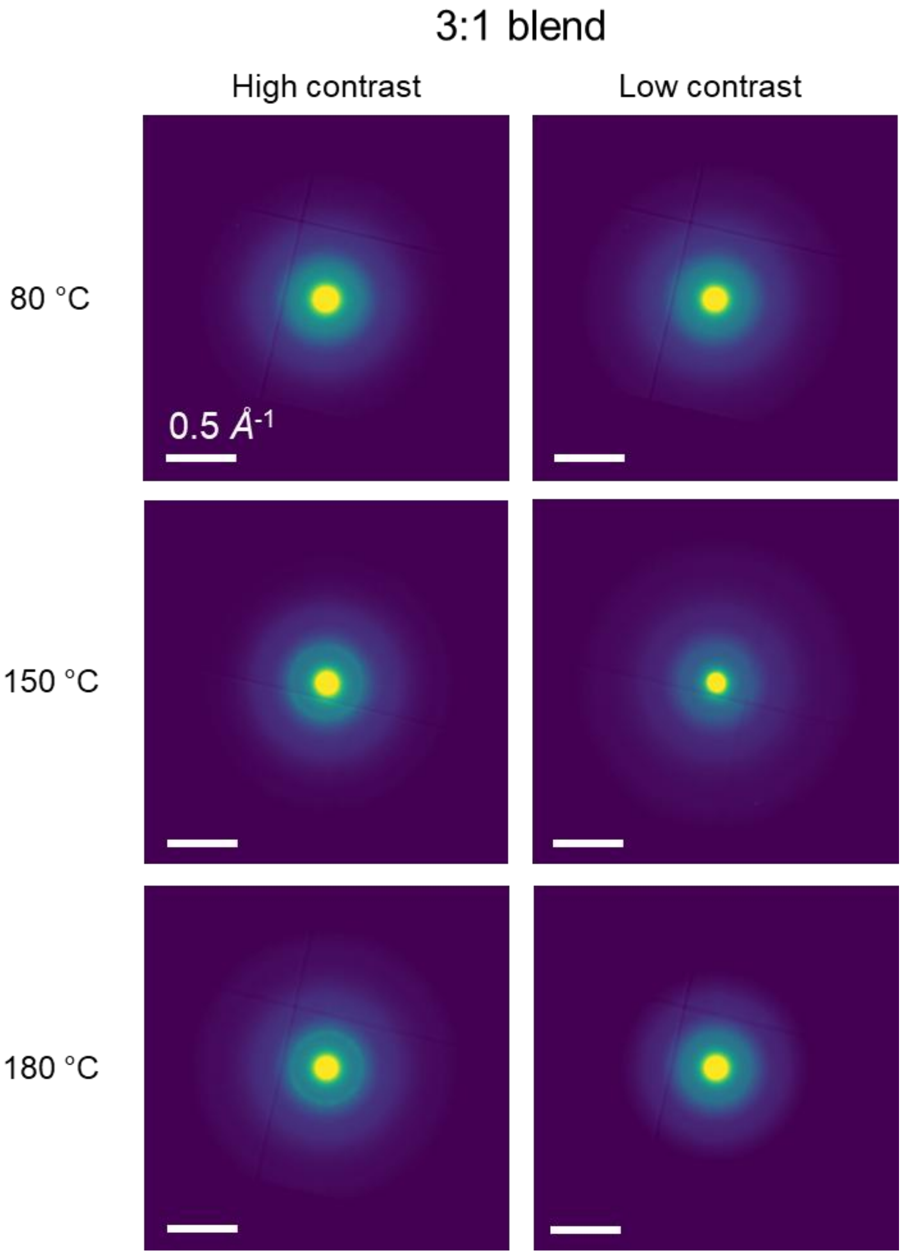

Figure S1. Average electron diffraction patterns integrated across high- and low-contrast areas in F8:F8BT (3:1) blend films. Diffraction patterns are shown for films prepared with heat treatment only at 80 °C as well as films with additional heat treatments at 150 °C or 180 °C.

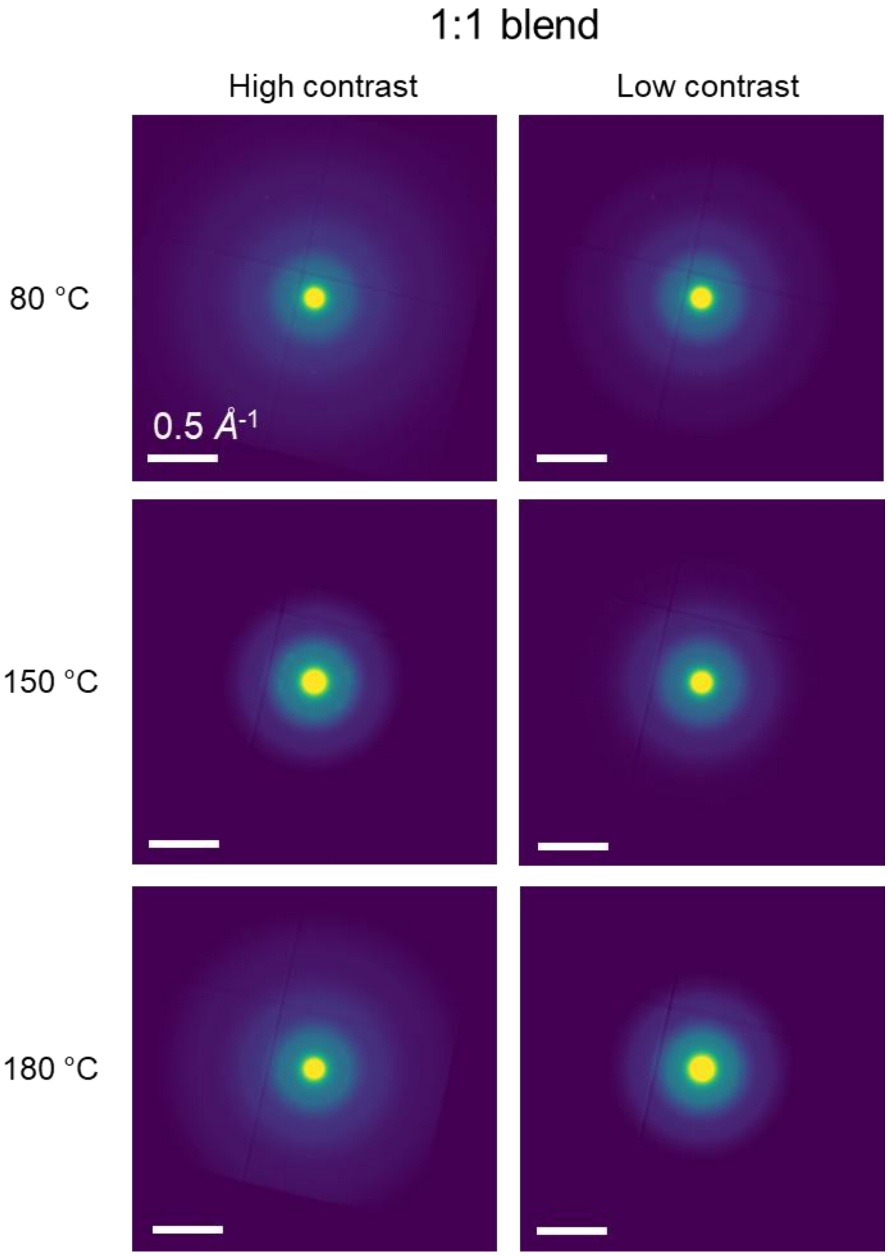

Figure S2. Average electron diffraction patterns integrated across high- and low-contrast areas in F8:F8BT (1:1) blend films. Diffraction patterns are shown for films prepared with heat treatment only at 80 °C as well as films with additional heat treatments at 150 °C or 180 °C.

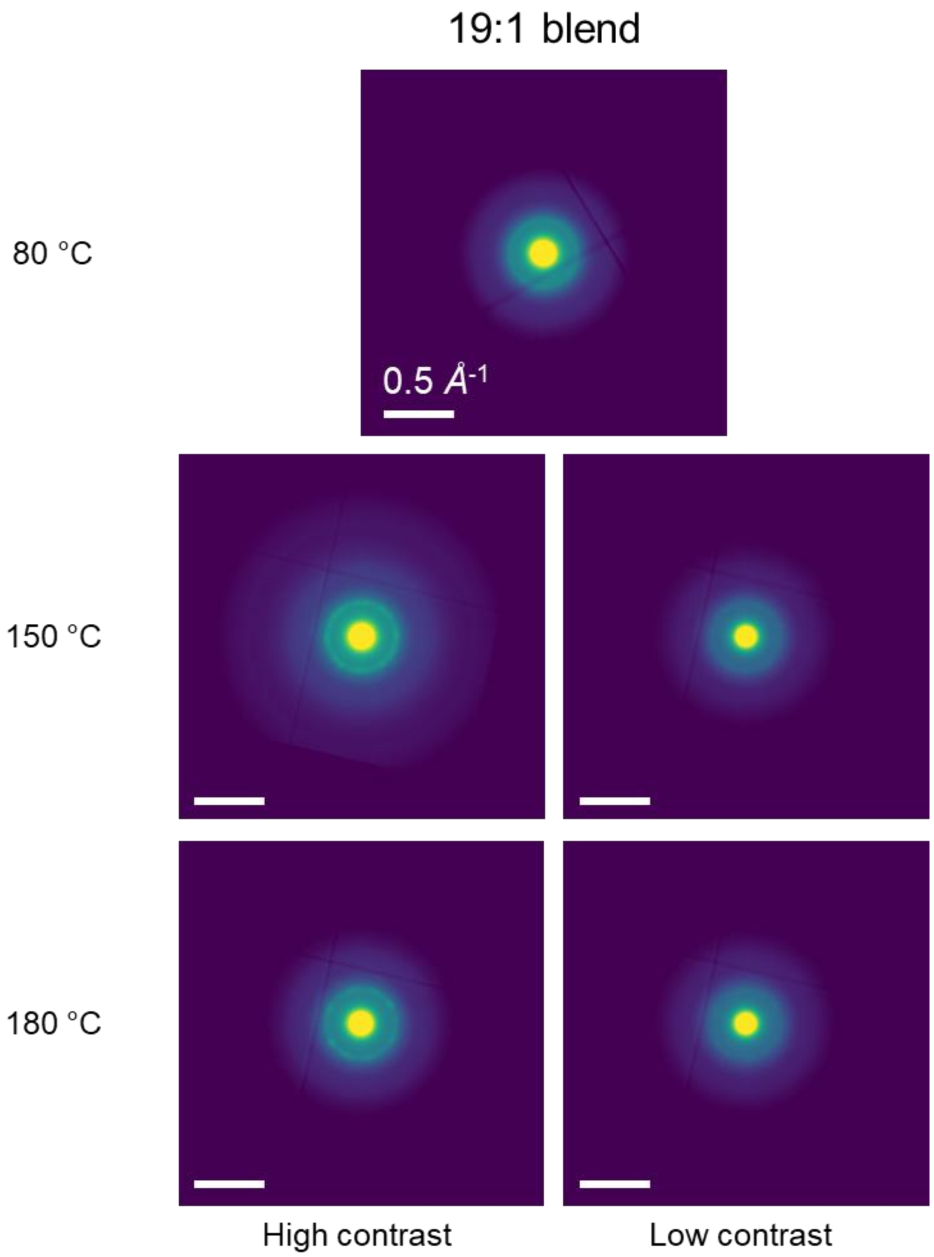

Figure S3. Average electron diffraction patterns integrated across (top row) the entire field of view and (middle and bottom rows) high- and low-contrast areas in F8:F8BT (19:1) blend films. Diffraction patterns are shown for films prepared with heat treatment only at 80 °C as well as films with additional heat treatments at 150 °C or 180 °C.

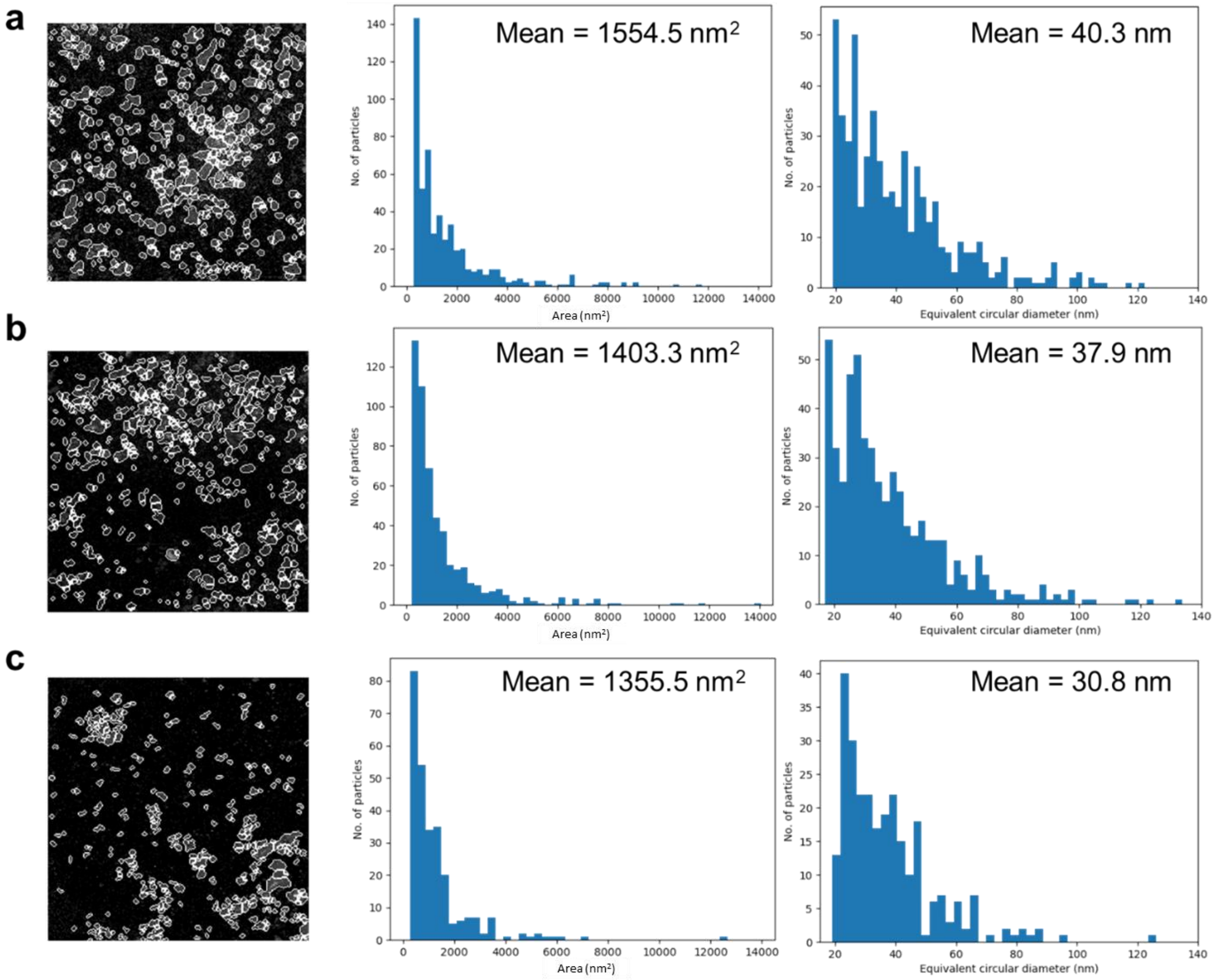

Figure S4. Segmentation of crystalline domains and corresponding histograms of the area and equivalent circular diameter of the crystalline domains for (a) 19:1, (b) 3:1, and (c) 1:1 (wt./wt.) blends after annealing at 150 °C.

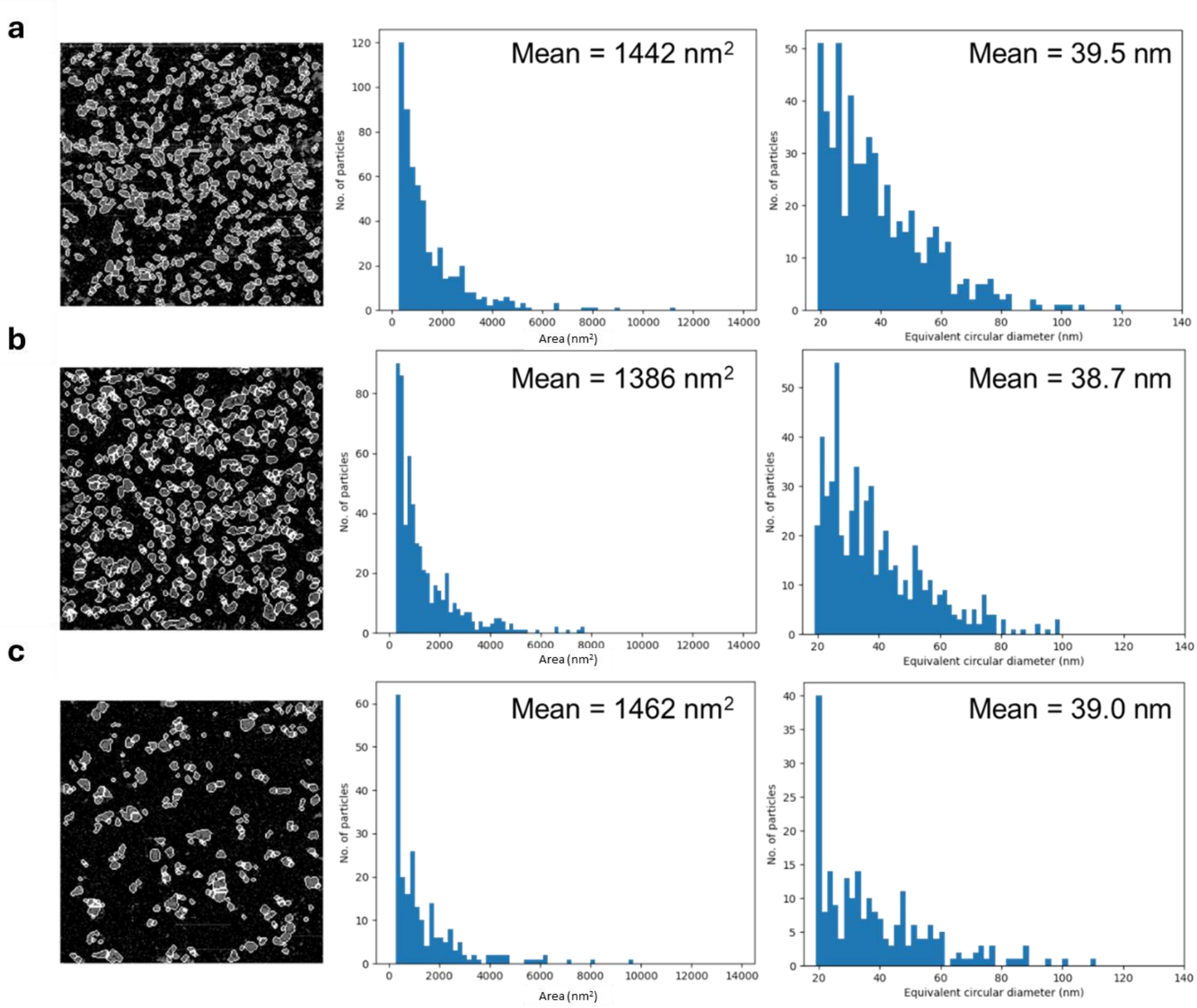

Figure S5. Segmentation of crystalline domains and corresponding histograms of the area and equivalent circular diameter of the crystalline domains for (a) 19:1, (b) 3:1, and (c) 1:1 (wt./wt.) blends after annealing at 180 °C.

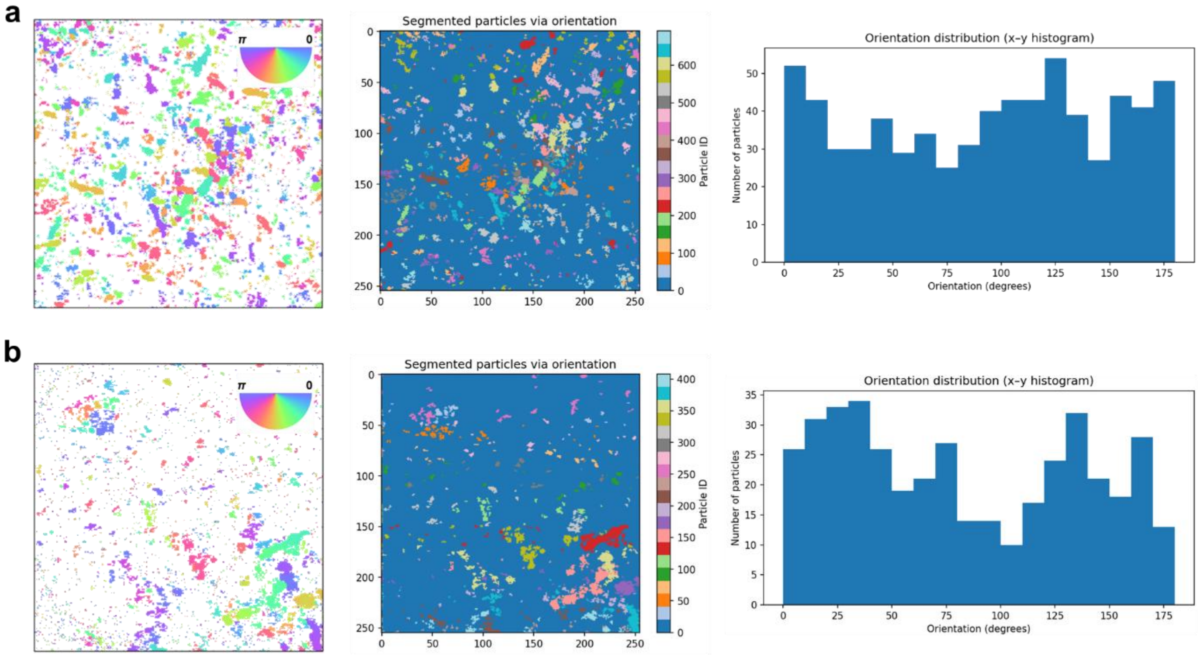

Figure S6. Orientation mapping and segmentation of crystalline domains for (a) 19:1 and (b) 1:1 (wt./wt.) blends annealed at 150 °C. The histogram shows the number of particles via orientation.

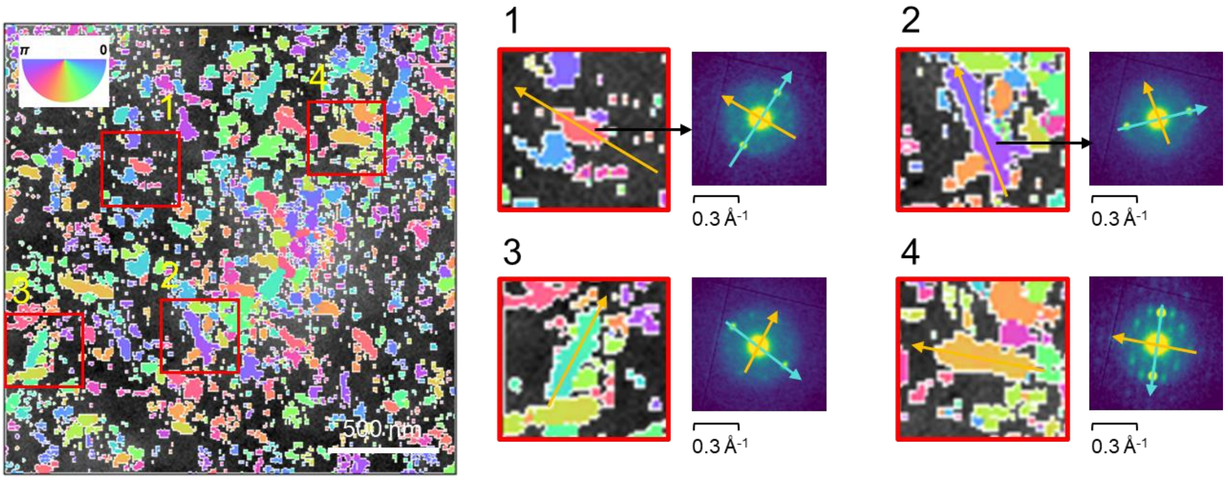

Figure S7. Selected areas in 19:1 (wt./wt.) blend annealed at 150 °C showing elongated particles and their corresponding diffraction patterns.

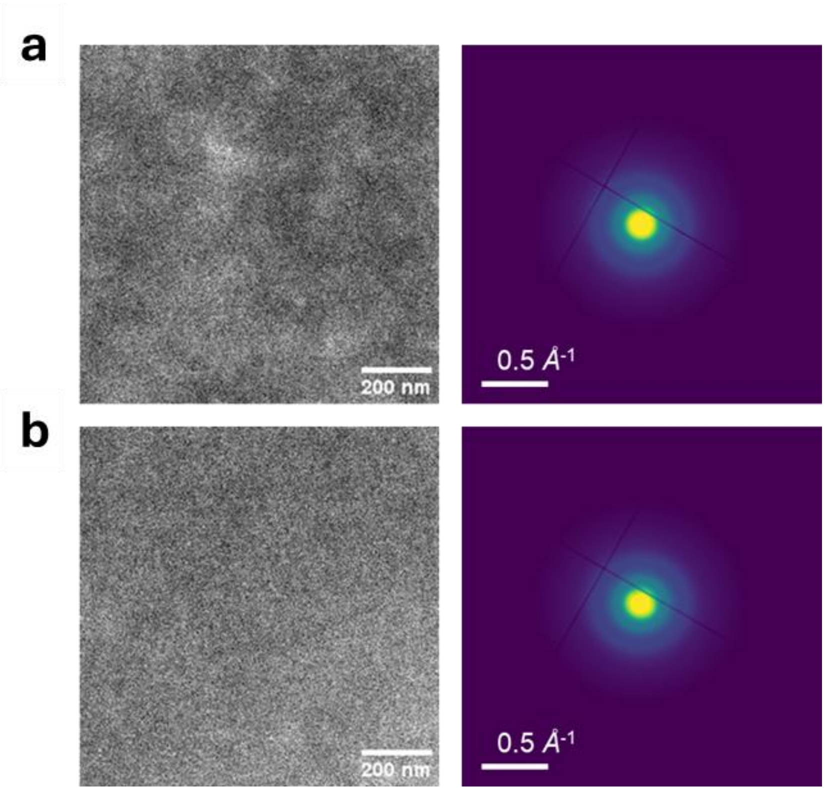

Figure S8. ADF images and average diffraction patterns of non-annealed (a) F8 and (b) F8BT polymer films.

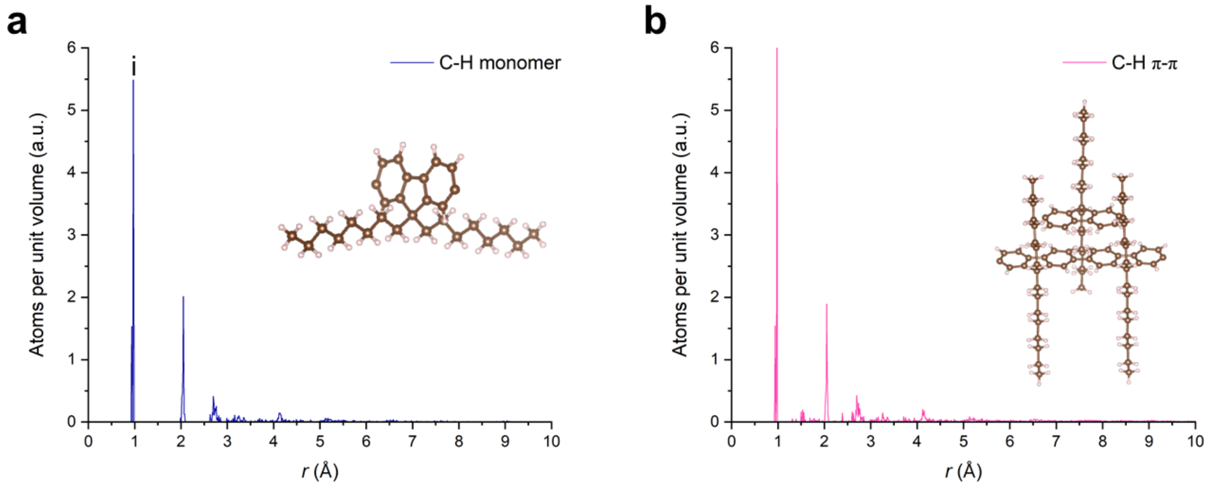

Figure S9. Distributions of C-H interatomic distances in (a) a single F8 monomer and (b) a model comprising three F8 monomers including $\pi - \pi$ stacking.

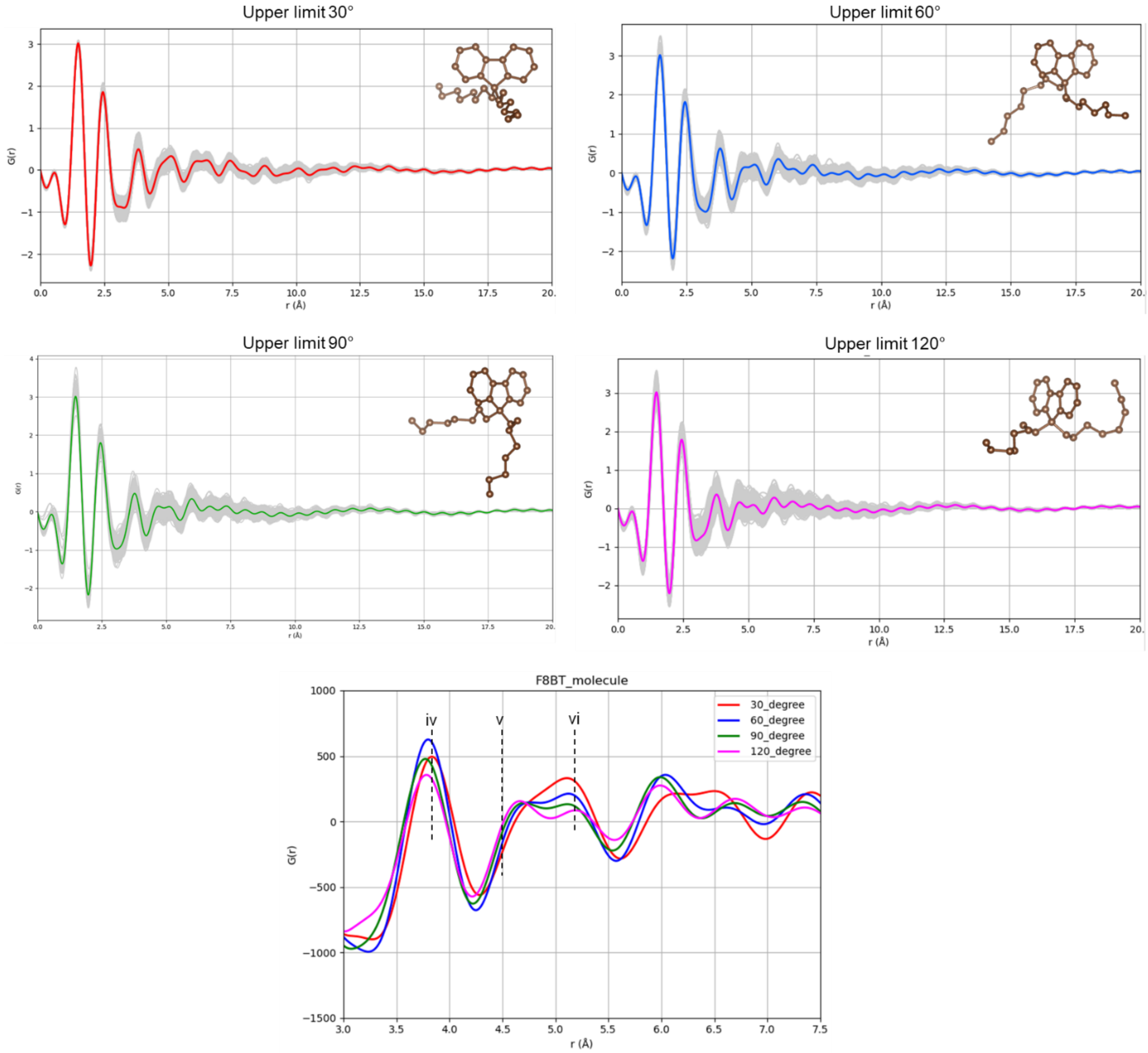

Figure S10. Calculated electron pair distribution function (ePDF) profiles for F8 monomers with varying upper-limit rotations of alkyl chains (30°, 60°, 90°, and 120°). The zoomed-in section of *r* from 3.0-7.5 Å shows the changes in peak position of (iv) and (vi) when varying the rotation of the alkyl chain. The peak at (v) does not emerge for all rotation degrees, suggesting that the peak (v) is more reasonably attributed to $\pi - \pi$ stacking.

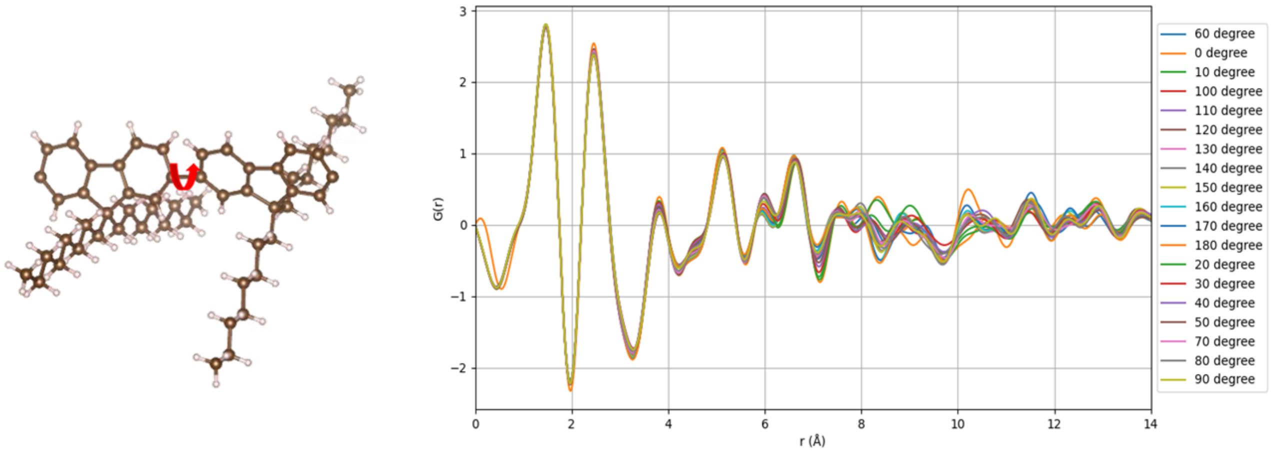

Figure S11. Calculated ePDF profiles for F8 dimers with varying relative rotations between fluorene backbones (0–180°).

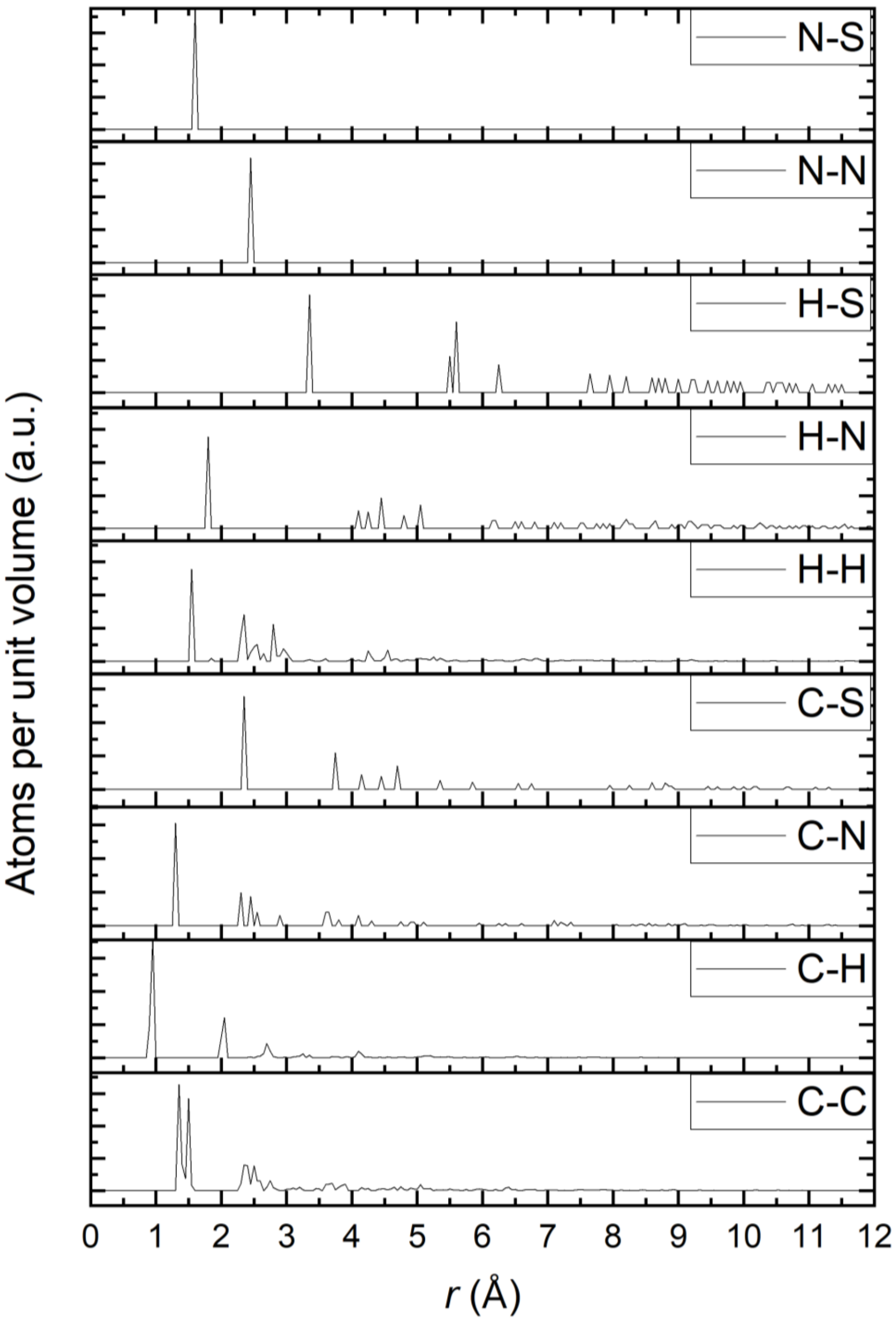

Figure S12. Distributions of all interatomic distances in a single F8BT monomer shown as element-wise pairs.

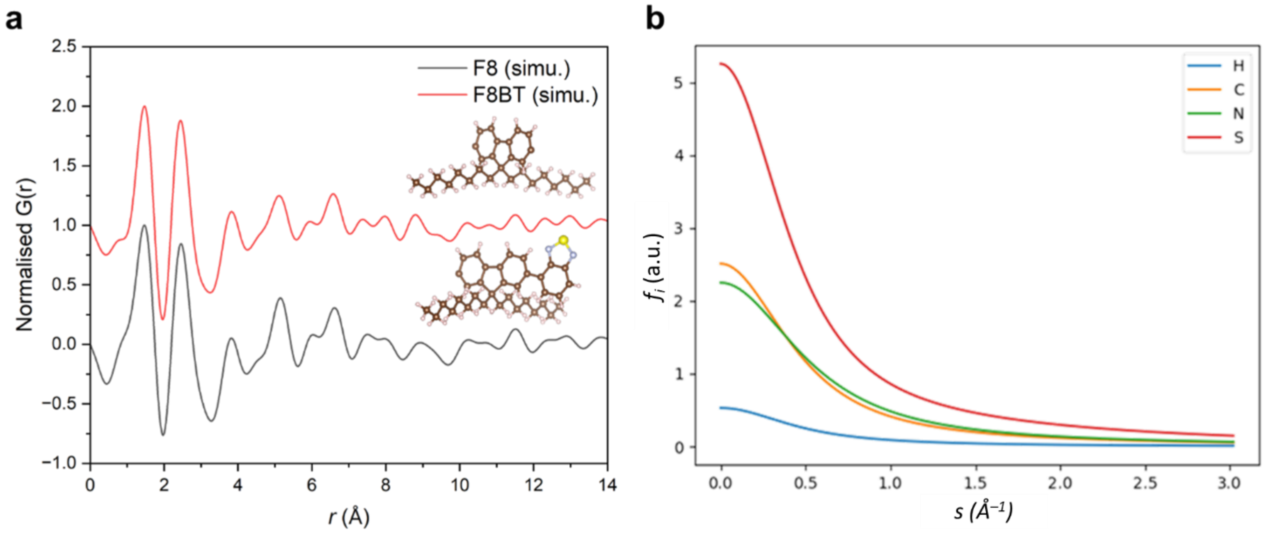

Figure S13. (a) Calculated ePDF profiles of F8 and F8BT monomers. (b) Calculated electron atomic scattering factors for hydrogen (H), carbon (C), nitrogen (N), and sulfur (S).

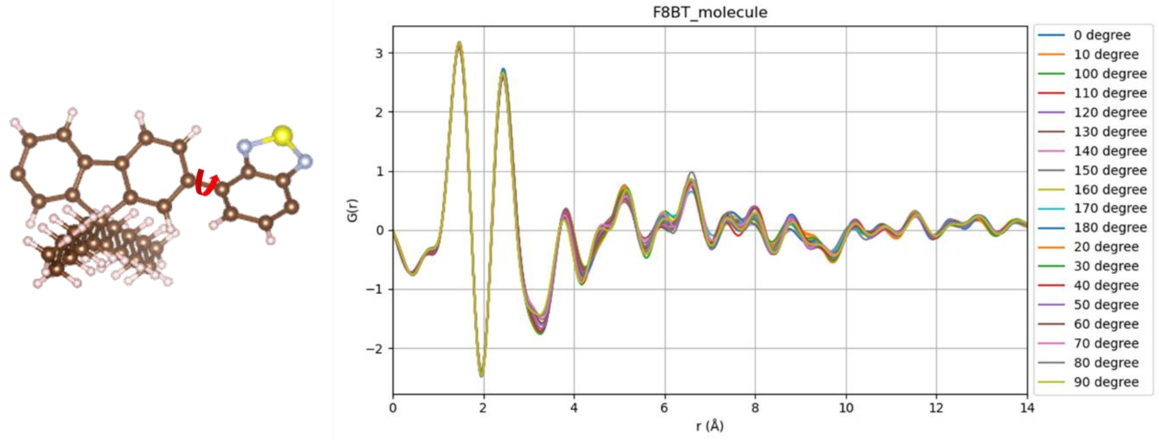

Figure S14. Calculated ePDF profiles for an F8BT monomer with varying relative rotations between fluorene backbone and benzothiadiazole moiety (0–180°).

Note on multivariate analysis of 1:1 blend ePDF results

Multivariate analysis has proven effective for unmixing the contributions of individual components in complex mixtures when using ePDFs, as demonstrated in systems such as organic semiconductor blends,[1] thermal barrier coatings,[2] and inorganic-organic glass mixtures.[3] Among the techniques used for multivariate analysis, a linear matrix decomposition using Principal Component Analysis (PCA) is widely used for processing hyperspectral data due to its ability to extract features associated with high variance. In some workflows, Independent Component Analysis (ICA) blind source separation is applied on the extracted PCA components to further isolate statistically independent source signals. For ePDF datasets, PCA is typically performed directly on the PDF profiles rather than reduced intensity profiles, as the former are less affected by variations in sample thickness.[3] PCA is advantageous because it minimizes subjective bias, enables rapid analysis of large datasets, and reveals dominant structural features. However, it may not always fully disentangle overlapping signals and does not inherently guarantee physical interpretability.

The PCA approach works by projecting the data onto an orthogonal basis set determined through simultaneous maximization of variance and minimization of projection error. Each basis vector, or principal component, is associated with an eigenvalue that reflects the proportion of variance it captures. The first few components usually account for the largest proportion of the total variance in the data, while subsequent ones represent less prominent variations. When applied to PDF data, meaningful components often reflect atom–atom correlations, while others may capture distortions or noise within the data.[4] Components that resemble the typical form of a PDF represent atom–atom correlations within the dataset. Components that deviate from the typical form of a PDF describe structural distortions of the atomic structure. While components that are dominated by high-frequency signals typically represent noise in the data. To ensure meaningful results, the interpretation of PCA should be guided by certain physical expectations, as set out by Chapman *et al.*[4]

PCA was applied to the F8:F8BT (1:1) blend dataset (Figure S15a), yielding a scree plot (Figure S15b) that illustrates the proportion of variance explained by the first 50 principal components. The inflection point or ‘elbow’ in the scree plot, indicated by the dashed line, suggests that at most three components are significant. The first principal component shows no distinctive features in its loading map (Figure S15c), and its corresponding factor spectrum shows a sharp, non-physical peak at r = 0.67 Å. This peak is likely an artefact arising from the transformation of reduced intensity data to ePDF. The second principal component has its corresponding factor spectrum resembling the typical form of an average ePDF, indicating it captures common atom–atom correlations within the data (Figure S15c). However, its loading map does not reveal any distinct spatial distribution indicative of phase-separated morphology. Instead, the features observed are likely artefacts arising from the centering transformation applied during post-processing of the 4D-STEM dataset. The third principal component also lacks discernible structure in its loading map, and its factor spectrum does not

exhibit characteristics typical of ePDF profiles. From the results, these findings suggest that PCA was ineffective at separating the signals from F8 and F8BT in this blend. This limitation is likely due to two key factors: (1) incomplete phase separation within the blend and (2) the high degree of similarity in the ePDF signatures of the two components.

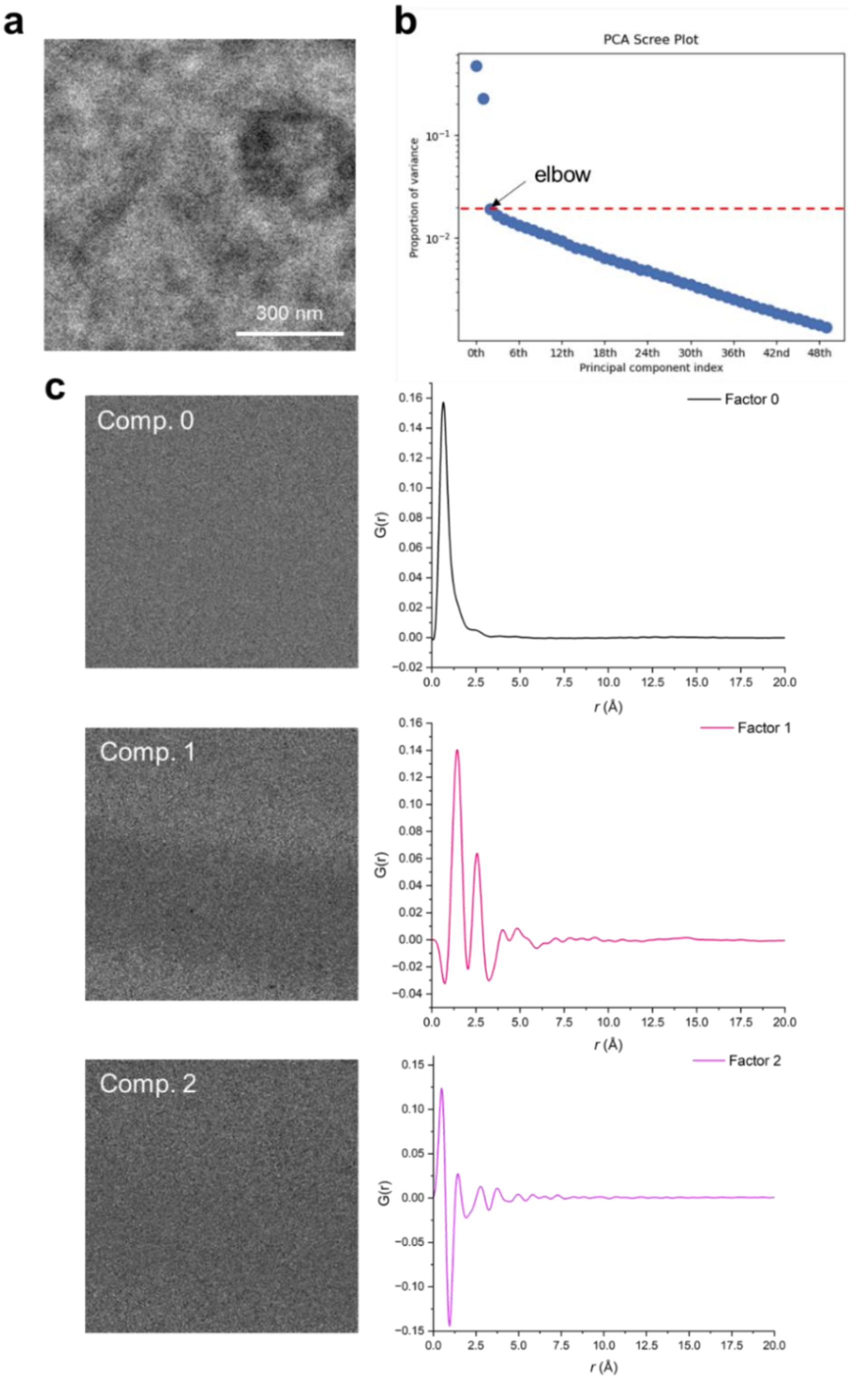

Figure S15. (a) ADF image of the F8:F8BT (1:1) blends without heat treatment. (b) PCA scree plot showing the proportion of variance associated with each of the first 50 principal components for the ePDFs extracted from the blend shown in (a). The 'elbow' in the scree plot, as marked by the dashed line, was used to estimate the number of factors that significantly contribute to the dataset. In this instance, three principal components were indicated by the scree plot. (c) Loading maps and corresponding spectral factors determined from PCA of ePDF data shown in (a).

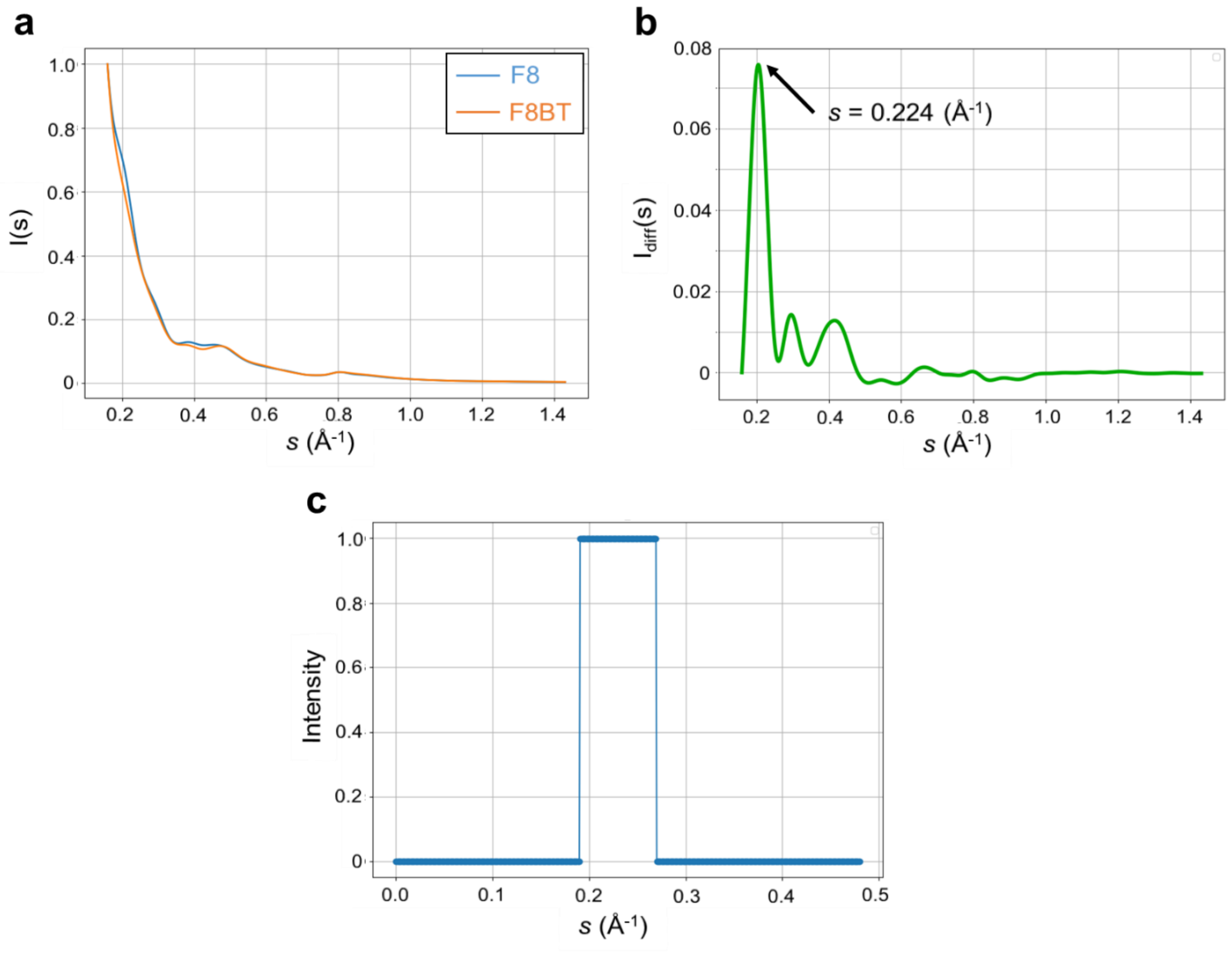

Figure S16. (a) Calculated scattering intensity profile of F8 and F8BT monomers. (b) Differential scattering intensity profile highlighting the peak contrast between F8 and F8BT, obtained by subtracting the F8 profile from the F8BT profile. (c) The top-hat function convolved with the differential scattering intensity profile.

Note on EELS analysis of phase separation in 1:1 blends

STEM-EELS hyperspectral mapping was performed on F8:F8BT (1:1) blends, both with and without heat treatment (Figure S17 and S18). The spatial distribution of F8-rich and F8BT-rich domains was inferred by comparing sulfur (S) mapping to thickness mapping. As an inelastic scattering-based technique, STEM-EELS can potentially induce radiation damage in polymer blends. The electron beam transfers energy to the sample predominantly through inelastic interactions, which can lead to chemical alterations, such as ionization or bond cleavage.[5] Depending on the degradation mechanism, these effects may result in either the formation of new bonds (e.g. cross-linking) or bond dissociation (via a gradual decrease in core-loss intensity of chemical elements).[6] The main target when performing STEM-EELS on polymers is to set the conditions where we can achieve a statistically significant signal-to-noise ratio. To achieve that, the probe current (measured on the samples) and the pixel acquisition time were examined in this study. As such, we selected the conditions that resulted in the estimated electron fluence of ~4.7×10^3 e^- $Å^{-2}$ per pixel. This experimental setup allows the observation of a sufficient signal-to-noise ratio for sulfur L_{23} edge while the morphology of phase separation in the polymer blends remains intact (Figure S17a and S18a), suggesting the preservation of the phase-separated morphology. The recorded EELS spectra at Area 1 and Area 2, as marked in Figure S17a, exhibit the characteristic sulfur L_{23} core-loss edge at 165 eV (Figure S17c and S18c).[7] The broad, featureless and rounded profile – dominated by atomic effects rather than solid-state electronic band structure – shows a delayed maximum approximately 20 eV beyond the ionization threshold. This spectral shape is typical for sulfur in polymer matrices and suggests that the F8BT component remained chemically stable under the applied electron fluence.

HAADF and EELS sulfur L_{23} edge mapping of F8:F8BT (1:1) polymer blends, with and without thermal treatment (Figures S17a–b and S18a–b), reveal that sulfur is more concentrated at the high contrast areas (i.e., the protruding domains) in the phase-separated morphology. This observation is unexpected, as previous studies have consistently shown that these protruding regions are enriched in F8,[8] which contains little to no sulfur. The apparent enrichment of sulfur in these areas, therefore, raises questions about the interpretation of the EELS sulfur L_{23} signal.

Two key factors may account for this observation. First, phase separation in the F8:F8BT (1:1) blend produces local variations in sample thickness between F8-rich and F8BT-rich regions, as evidenced by the EELS thickness maps (Figures S17b and S18b). Second, the phase separation is incomplete, resulting in partial intermixing between the two components; thus, F8BT is present within F8-rich domains and vice versa. Although the precise composition of each domain cannot be quantified from the present data, it is reasonable to hypothesize that F8-rich regions may contain a greater absolute amount of F8BT than the F8BT-rich regions, simply due to their greater local thickness, approximately twice as thick, as shown in the t/λ maps (Figures S17b and S18b), which provides more volume to accommodate material. This thickness-dependent effect is supported by the non-

annealed sample. When the exported EELS spectra from regions labelled as Area 1 and Area 2, corresponding to high and low apparent sulfur intensity (Figure S17b), were processed by Fourier-ratio deconvolution (to remove plural scattering) and normalized to the carbon *K* edge, negligible differences in sulfur L_{23} intensity were observed (Figure S17c). This indicates that the apparent sulfur variation arises mainly from thickness differences rather than compositional variation. In contrast, the annealed sample exhibits a genuine increase in sulfur signal intensity in the high-contrast regions (Area 2) (Figure S18c), suggesting a higher local F8BT content compared to the low-contrast areas (Area 1). This implies that, although the low-contrast domains may be richer in F8BT on a relative basis, their total F8BT content remains lower than that of the thicker, F8-rich protruding regions. Notably, while the EELS t/λ and sulfur L_{23} maps correlate well in the non-annealed blend (Figure S17b), distinct deviations appear after annealing (Figure S18b). In certain areas highlighted by dashed black ellipses, the sulfur intensity decreases despite similar local thickness. These protruding domains, which show reduced sulfur L_{23} signal relative to neighboring regions, likely contain a higher proportion of F8. Comparison with the corresponding HAADF images (Figure S18a) reveals that these domains coincide with the areas where nanocrystalline features attributed to F8 formation upon annealing appear, as discussed in Figure 2. Overall, the EELS analysis provides consistent evidence supporting the conclusion that the nanocrystalline domains observed in the annealed blends correspond to F8 nanocrystals.

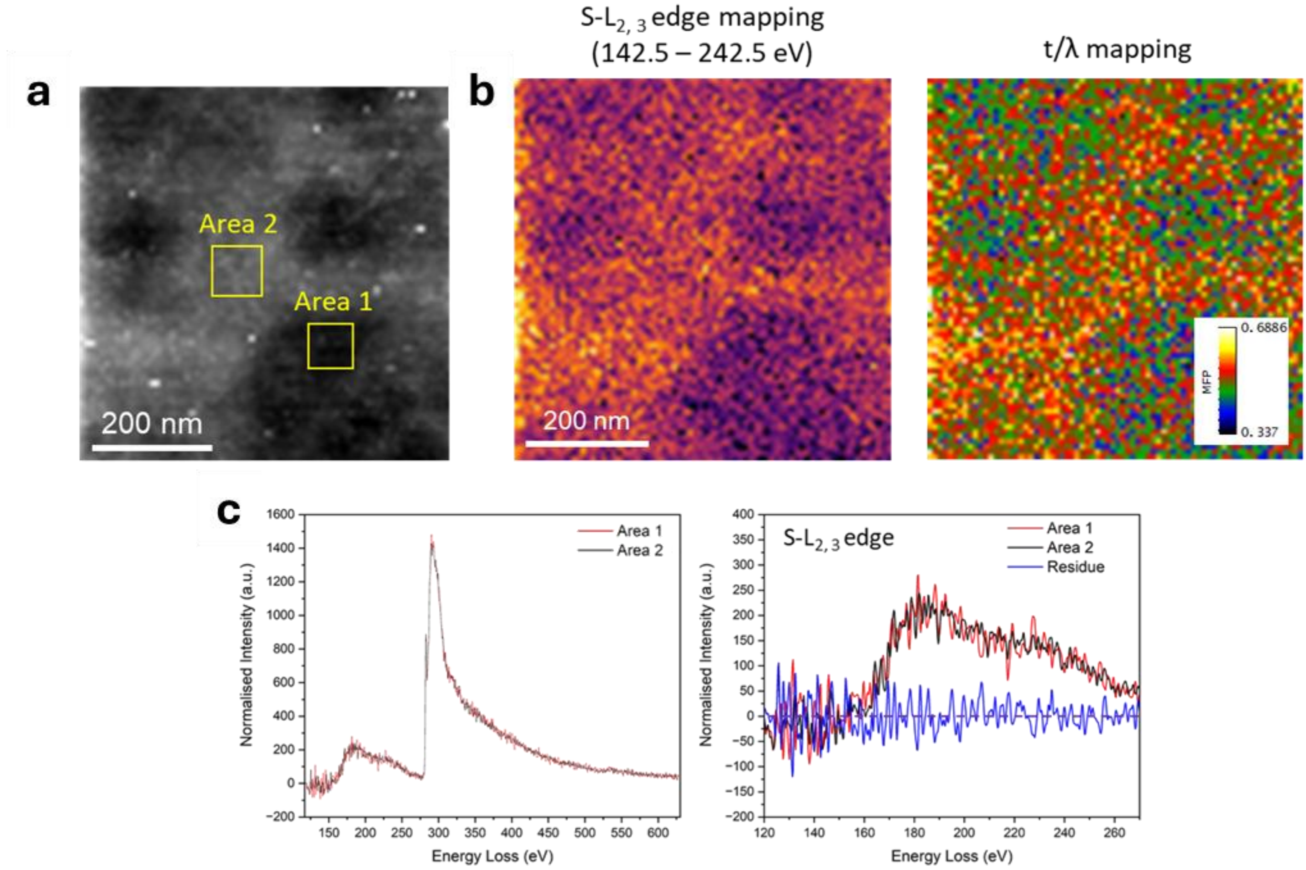

Figure S17. (a) HAADF image of the phase-separated morphology in F8:F8BT (1:1) blend without heat treatment. (b) Mapping of sulfur distribution, using sulfur L_{23} energy loss edge, and t/λ in the phase-separated morphology. (c) Overview and sulfur L_{23} energy loss spectrum extracted from the high-sulfur and low-sulfur areas, with the residual showing the difference between the signals between the two areas. The energy core loss spectra are already deconvolved using Fourier ratio deconvolution to remove the effects of plural scattering and normalized using the carbon K edge at both π* and σ* peaks.

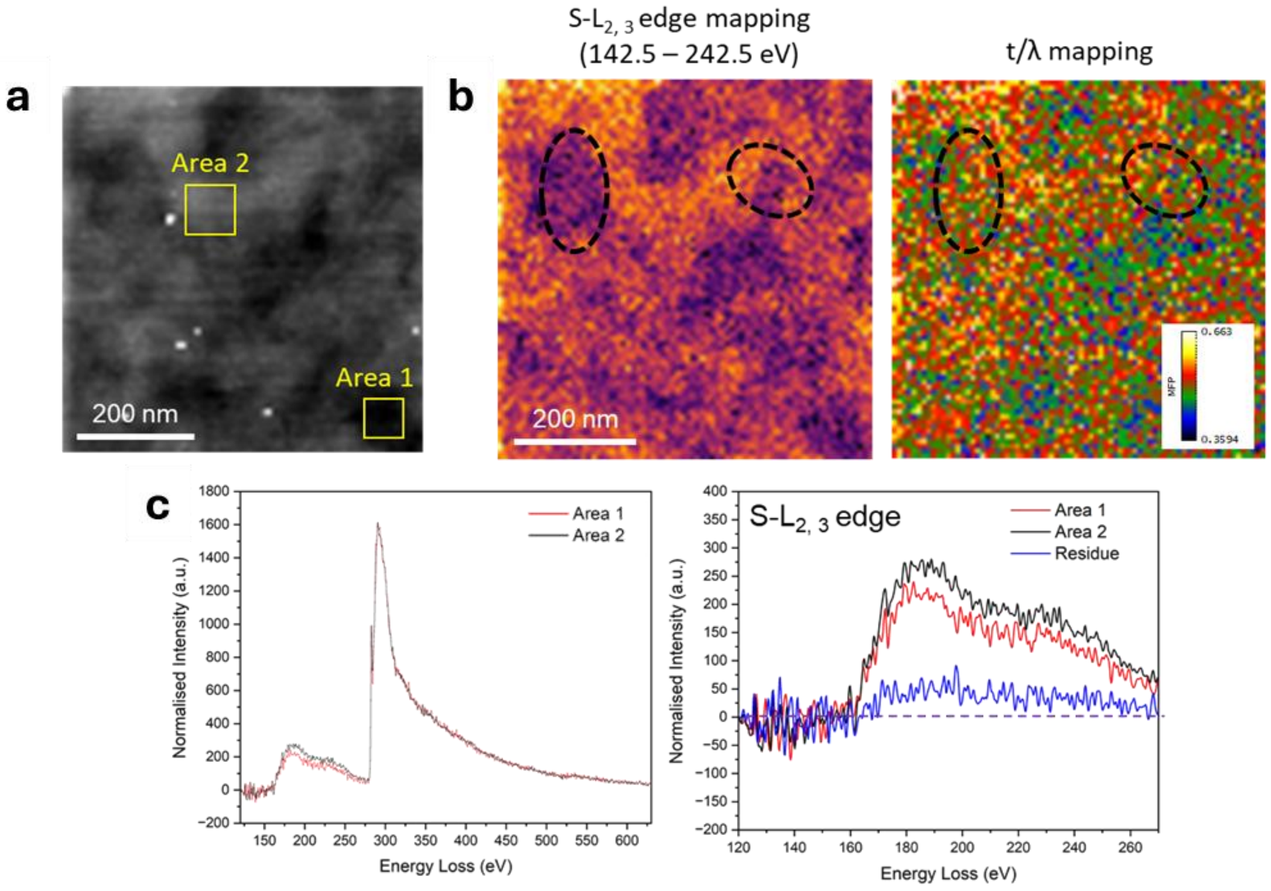

Figure S18. (a) HAADF image of the phase-separated morphology in F8:F8BT (1:1) blend annealed at 150 °C. (b) Mapping of sulfur distribution, using sulfur L_{23} energy loss edge, and t/λ in the phase-separated morphology. The black, dashed ellipses mark the areas where there is no correlation between the thickness and sulfur intensity variations. (c) Overview and sulfur L_{23} energy loss spectrum extracted from the high-sulfur and low-sulfur areas, with the residual showing the difference between the signals between the two areas. The energy core loss spectra are already deconvolved using Fourier ratio deconvolution to remove the effects of plural scattering and normalized using the carbon *K* edge at both π* and σ* peaks.

Note on evaluation of FIB-induced damage by SED-ePDF

In addition to phase analysis and crystallinity mapping, we note that 4D-STEM-ePDF might allow the assessment of the ion beam-induced damage on the polymer layers. For molecular materials, the nearest neighbor atomic distances ($r < 2$ Å) in ePDF can be used to evaluate the chemical bonding of an individual molecule.[9] Figure S19 shows the ePDF profile extracted from the amorphous and crystalline areas in the F8:F8BT blend layer in the cross-sectioned model device in comparison to the ePDF profile extracted from the corresponding plan-view films. There is a good alignment for pairwise atomic distances at $2 < r < 6$ Å between ePDFs extracted from plan-view films, amorphous and crystalline regions in cross-sectioned device. However, the nearest neighbor atomic distance ($r < 2$ Å) shows a distinguishable shift for the ePDF profile of the amorphous region in the cross-sectioned device compared to those from the plane view films and crystalline region (Figure S17b). The shift from $r = 1.49$ Å to $r = 1.42$ Å ($\Delta r = 0.07$ Å) for the nearest neighbor atomic distance could suggest a disappearance of chemical bonds within $r = 1$–2 Å due to ion beam-induced heat damage; for example, the disappearance of N-S with $r = 1.6$ Å could shift the first nearest neighbor atomic distance to lower r given that this atomic distance in pair-wise distribution is the convolution of multiple peaks that represent chemical bonding in the intramolecular structure.[9] We note that the shift in r of 0.07 Å is much smaller than the resolution defined by the effective Q_{max} (9 $Å^{-1}$, $\Delta r = 0.42$ Å) or the Q_{max} set by the detector configuration ($Q_{max} = 19$ $Å^{-1}$, $\Delta r = 0.20$ Å). While precision in peak positions can be much greater than the resolution required to distinguish two adjacent peaks, the interpretation of peak shifts cannot be unambiguously attributed to loss of N-S bonding originating in FIB damage due to the variety of effects contributing to the ePDF profile.

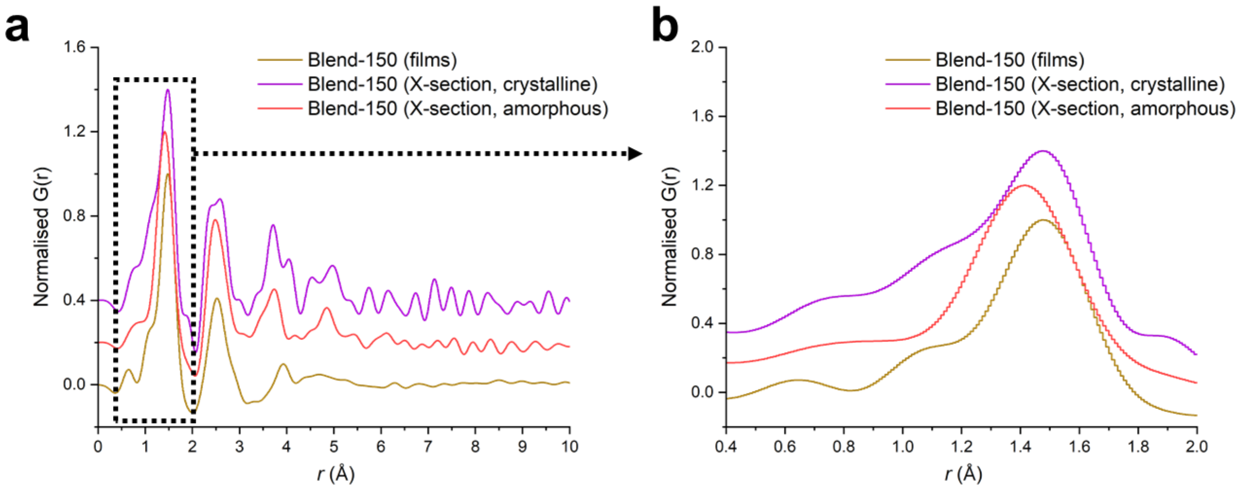

Figure S19. (a) ePDF profiles of the F8:F8BT (1:1) blend annealed at 150 °C extracted from the plane-view films, amorphous regions, and crystalline regions in the cross-sectioned device model. (b) Enlarged view of the first peak in the ePDF profiles showing a distinguishable shift toward the lower interatomic distance *r* for the amorphous regions in the cross-sectioned device model.

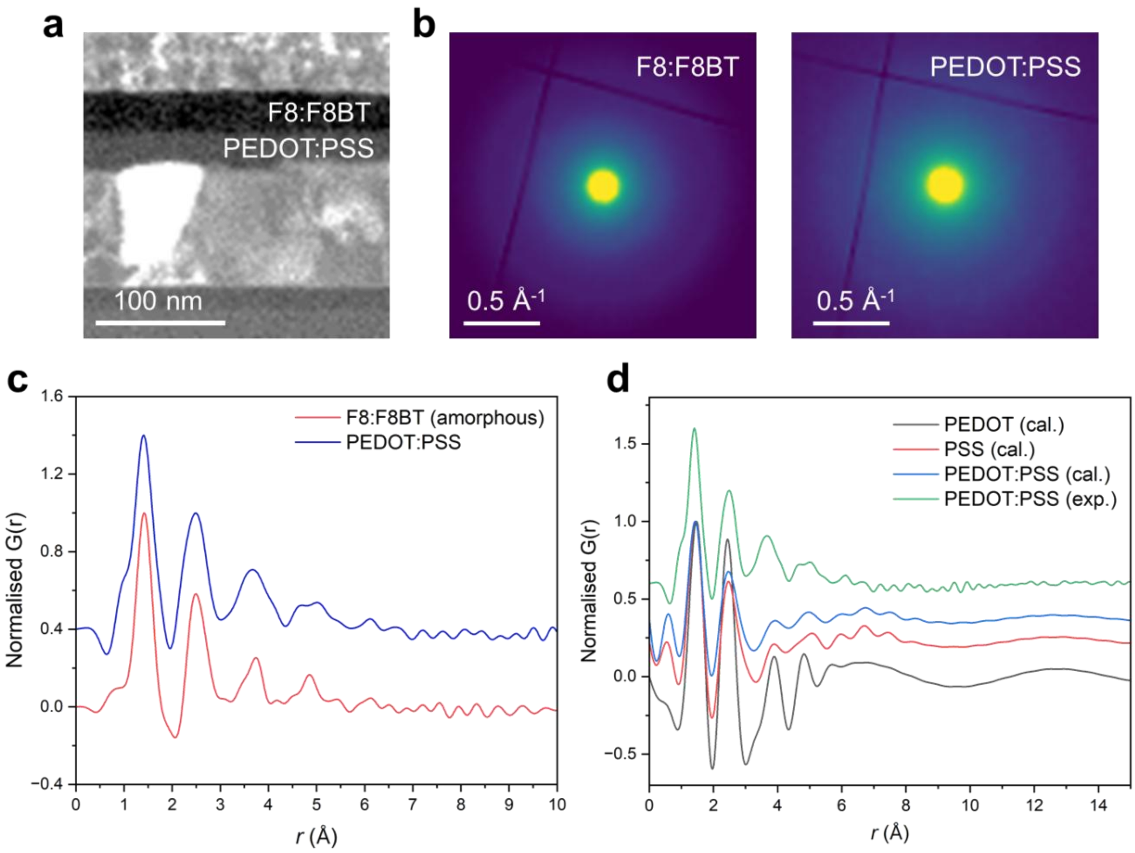

Figure S20. (a) ADF image of the cross-sectioned OLED model device showing distinct contrast between F8:F8BT (1:1) and PEDOT:PSS (1:6) layers. (b) Average diffraction patterns isolated from the F8:F8BT (1:1) and PEDOT:PSS (1:6) layers. (c) ePDF profiles obtained from the average diffraction pattern of F8:F8BT (1:1) and PEDOT:PSS (1:6) layers. (d) Comparison between the calculated ePDF profiles for PEDOT, PSS, PEDOT:PSS (1:6) and experimental PEDOT:PSS (1:6).

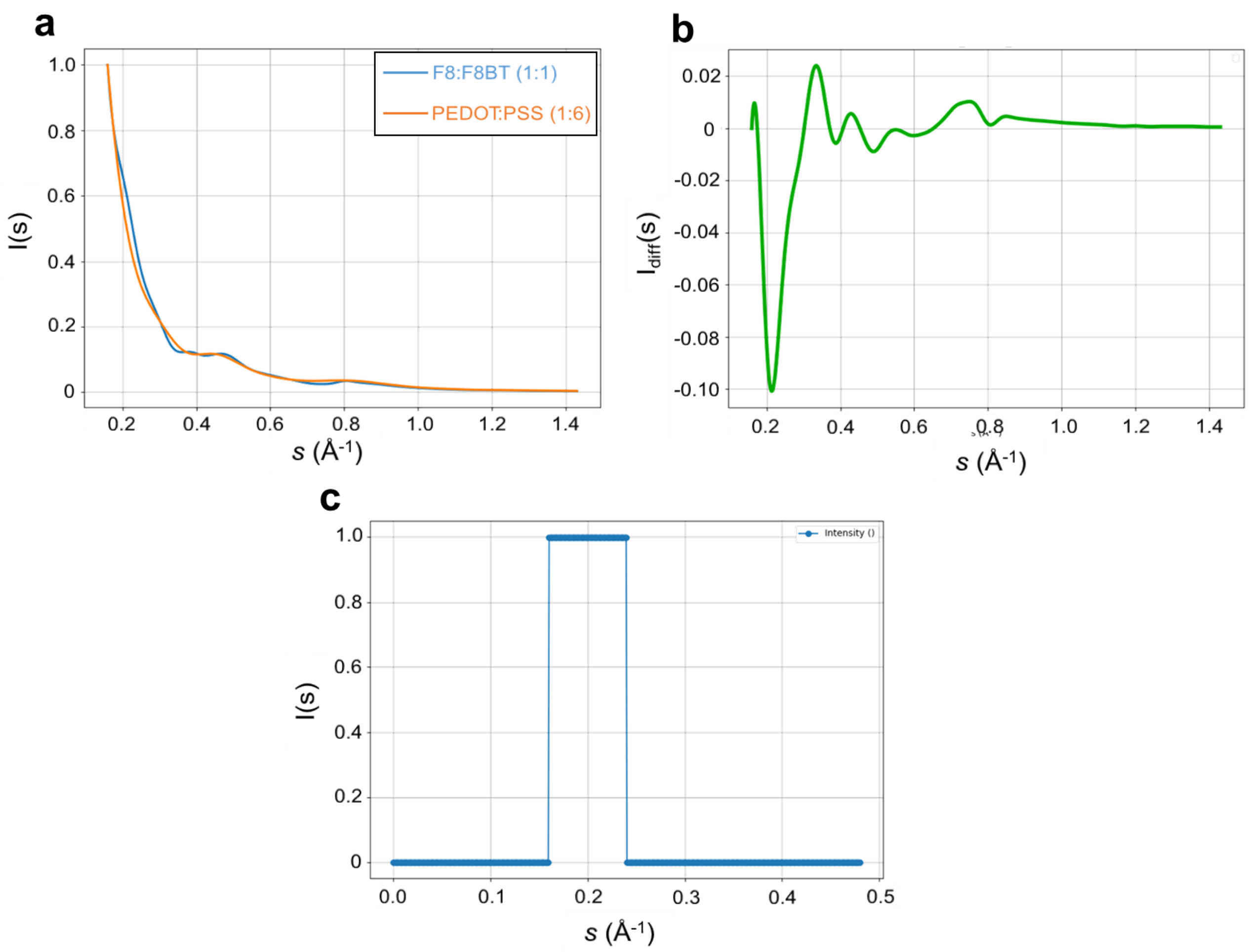

Figure S21. (a) Calculated scattering intensity profile of F8:F8BT (1:1) and PEDOT:PSS (1:6) blends. (b) Differential scattering intensity profile highlighting the peak contrast between two blends, obtained by subtracting the PEDOT:PSS (1:6) profile from the F8:F8BT (1:1) profile. (c) The top-hat function convolved with the differential scattering intensity profile.

References

[1] X. Mu, A. Mazilkin, C. Sprau, A. Colsmann, C. Kübel, *Microscopy* **2019**, *68*, 301.
[2] X. Mu, D. Wang, T. Feng, C. Kübel, *Ultramicroscopy* **2016**, *168*, 1.
[3] J. E. M. Laulainen, D. N. Johnstone, I. Bogachev, L. Longley, C. Calahoo, L. Wondraczek, D. A. Keen, T. D. Bennett, S. M. Collins, P. A. Midgley, *Nanoscale* **2022**, *14*, 16524.
[4] K. W. Chapman, S. H. Lapidus, P. J. Chupas, *J. Appl. Crystallogr.* **2015**, *48*, 1619.
[5] R. Pal, A. K. Sikder, K. Saito, A. M. Funston, J. R. Bellare, *Polym. Chem.* **2017**, *8*, 6927.
[6] L. M. Valencia, M. de la Mata, M. Herrera, F. J. Delgado, J. Hernández-Saz, S. I. Molina, *Polym. Degrad. Stab.* **2022**, *203*, 110044.
[7] V. P. Oleshko, J. Kim, J. L. Schaefer, S. D. Hudson, C. L. Soles, A. G. Simmonds, J. J. Griebel, R. S. Glass, K. Char, J. Pyun, *MRS Commun.* **2015**, *5*, 353.
[8] R. Stevenson, R. Riehn, R. G. Milner, D. Richards, E. Moons, D.-J. Kang, M. Blamire, J. Morgado, F. Cacialli, *Appl. Phys. Lett.* **2001**, *79*, 833.
[9] M. W. Terban, S. J. L. Billinge, *Chem. Rev.* **2022**, *122*, 1208.
